# Polydopamine Nanobowl‐Armoured Perfluorocarbon Emulsions: Tracking Thermal‐ and Photothermal‐Induced Phase Change through Neutron Scattering

**DOI:** 10.1002/smll.202406019

**Published:** 2024-11-10

**Authors:** Mark Louis P. Vidallon, Haikun Liu, Zhenzhen Lu, Shahinur Acter, Yuyang Song, Chris Baldwin, Boon Mian Teo, Alexis I. Bishop, Rico F. Tabor, Karlheinz Peter, Liliana de Campo, Xiaowei Wang

**Affiliations:** ^1^ Molecular Imaging and Theranostics Laboratory Baker Heart and Diabetes Institute 75 Commercial Road Melbourne VIC 3004 Australia; ^2^ Baker Department of Cardiometabolic Health University of Melbourne Parkville VIC 3010 Australia; ^3^ School of Chemistry Monash University Clayton VIC 3800 Australia; ^4^ Baker Department of Cardiovascular Research Translation and Implementation La Trobe University Bundoora VIC 3086 Australia; ^5^ Department of Chemical Engineering University of Melbourne Parkville 3010 Australia; ^6^ Department of Radiation Oncology and Molecular Sciences The Johns Hopkins School of Medicine Johns Hopkins University 733 N Broadway Baltimore MD 21205 USA; ^7^ Australian Nuclear Science and Technology Organization (ANSTO) New Illawarra Rd Lucas Heights NSW 2234 Australia; ^8^ School of Physics and Astronomy Monash University Clayton VIC 3800 Australia; ^9^ Atherothrombosis and Vascular Biology Laboratory Baker Heart and Diabetes Institute 75 Commercial Road Melbourne VIC 3004 Australia; ^10^ School of Translational Medicine Monash University Melbourne VIC 3004 Australia

**Keywords:** near‐infrared, neutron scattering, phase‐change emulsions, pickering emulsion, polydopamine nanobowls

## Abstract

Anisotropic polydopamine nanobowls (PDA NBs) show significant promise in biomedicine, distinguished by their unique optical properties and superior cellular uptake compared to spherical nanoparticles. This study presents a novel approach for creating multistimuli‐activated PDA NB‐armored emulsions, encapsulating perfluorohexane (NB‐H) and perfluoropentane (NB‐P) cores, with applications in controlled delivery and ultrasound imaging. Thermal and photothermal activation induced distinct responses in the emulsions, as evidenced by optical microscopy and thermogravimetric analysis. For the first time, neutron scattering techniques (SANS and USANS) under contrast matching conditions are applied to investigate these materials, revealing detailed droplet and microbubble structures and phase transition dynamics. These results show that NB‐H droplets resist phase change under direct heating, whereas NB‐P droplets respond more readily, exhibiting significant bubble formation. During photothermal activation with short near‐infrared (NIR) exposure (15 min at 400 mW cm^−2^), SANS and USANS analyses reveal varying degrees of phase transition, proving this activation method to be more effective than direct heating. Importantly, NB‐H and NB‐P droplets have excellent ultrasound contrast enhancement and biocompatibility, indicating their potential for contrast‐enhanced ultrasound imaging, theranostics, and photothermal applications. This comprehensive study advances the understanding of multifunctional colloidal materials in biomedicine, contributing essential knowledge to this rapidly evolving field.

## Graphical abstract

Novel Pickering emulsions with perfluorocarbon (PFC) cores and polydopamine nanobowl (PDA NB) shells form a unique “armored” droplet. Combining droplet‐stabilizing and photothermal conversion properties of PDA NBs with the thermal adaptability and ultrasound backscattering of PFCs, these materials are highly versatile. Small‐and ultra‐small‐angle neutron scattering techniques reveal their thermal and photothermal responsiveness, showcasing their potential as next‐generation materials.

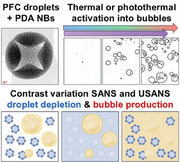

## 1 Introduction

The emergence of polydopamine (PDA) as a multifunctional colloidal material for a diverse range of biomedical applications can be attributed to its exceptional physical properties, in addition to its excellent biocompatibility and biodegradability.^[^ ^1^, ^2^, ^3^ ^]^ This nature‐inspired biopolymer undergoes polymerization in a basic environment through autoxidation of dopamine, a neurotransmitter in the central nervous system.^[^ ^3^ ^]^ PDA's distinctive optoelectronic properties enable it to absorb a broad spectrum of electromagnetic radiation, spanning from visible light to near‐infrared wavelengths. Consequently, the absorbed radiant energy is converted into heat and ultrasound (for pulsed sources), underscoring its potential in diverse biomedical applications, such as controlled drug release, ultrasound and photoacoustic imaging, photothermal ablation therapy, and combined chemo‐ and photothermal therapies.^[^ ^4^, ^5^, ^6^, ^7^ ^]^ Due to their surface activity and flexibility, PDA materials are key components for hybrid colloids and interfaces, offering unique structural features and optical properties that advance nanomedicine.

Among these morphological variants, PDA nanobowls (NBs) have gained considerable attention due to their unique anisotropic shapes, which provide enhanced particle uptake and distribution within biological systems. Unlike traditional spherical and isotropic nanoparticles, PDA NBs exhibit anisotropic shapes, presenting a novel paradigm for tailored applications. This anisotropy results in larger surface areas and increased contact points with target cells, improving cellular internalization and therapeutic efficiency,^[^ ^4^, ^8^, ^9^, ^10^, ^11^, ^12^ ^]^ which are key attributes for nanomaterials in drug delivery, diagnostics, and imaging applications.^[^ ^13^ ^]^ Moreover, their anisotropic shape can induce intriguing self‐assembly behaviors in colloidal systems, leading to unique optical and mechanical properties, which can be harnessed for innovative applications, particularly in the realm of multifunctional nanomedicine for cancer treatment.^[^ ^13^ ^]^

The amphiphilic nature of PDA NBs further enhances their utility in biomedical applications. Their unique amphiphilic properties enable them to interact with both hydrophobic and hydrophilic components, rendering them ideal candidates for stabilizing complex colloidal systems. PDA NBs serve as Pickering stabilizers, characterized by solid particles adsorbing at the oil‐water interface,^[^ ^14^, ^15^ ^]^ which are pivotal for drug delivery and therapeutic applications due to their enhanced stability and controlled release properties.^[^ ^16^ ^]^ For the first time, in 2021, our team introduced PDA NBs as Pickering stabilizers without any surface modification or contribution from other stabilizers.^[^ ^17^ ^]^ The resulting oil‐in‐water Pickering emulsion system demonstrated prolonged stability, pH responsiveness, and remarkable photothermal response under NIR exposure, showcasing the unique wetting thermodynamics of PDA NBs at oil–water interfaces. Leveraging the photothermal properties of PDA, this Pickering emulsion system exhibited a strong photothermal response, ideal for various biomedical applications, including NIR‐triggered drug delivery. Surprisingly, despite the apparent advantages that PDA NBs can offer as Pickering stabilizers, only a limited number of reports have explored their use in biomedical colloidal materials, highlighting a significant research gap and untapped potential of PDA NBs in this context.

Phase‐change emulsions represent a unique class of materials that have recently piqued interest in the biomedical field. These liquid core emulsions are composed of perfluorocarbons (PFCs), typically with low boiling points (Tb) close to body temperature (e.g., perfluoropentane, PFP with T_b_ = 30 °C) or with more thermally stable PFCs (e.g., perfluorohexane, PFH with T_b_ = 56 °C and perfluorooctyl bromide with T_b_ = 142 °C). These materials are designed to undergo either reversible or irreversible phase transitions into microbubbles, in response to external stimuli, with heat, ultrasound, photothermal induction, and magnetic heating being the most commonly used triggers.^[^ ^5^, ^18^, ^19^, ^20^ ^]^ Phase‐change emulsions have the potential to revolutionize drug delivery, imaging, and thermal therapy by capitalizing on their volume and density change upon activation, as well as the properties of their shell materials, typically drug cargo‐carrying and release capabilities, optical properties, and localized heat production within biological systems.^[^ ^5^, ^21^, ^22^ ^]^ Advanced applications of different phase‐change emulsion systems have also been demonstrated in recent works, including in super resolution imaging,^[^ ^23^ ^]^ neuromodulation,^[^ ^24^ ^]^ and sonothrombolysis.^[^ ^25^ ^]^

Our study introduces several key innovations in PDA applications and PFC technologies that distinguish it from previous works in the field. For the first time, we utilized PDA NBs, anisotropic, bowl‐shaped mesoporous PDA nanoparticles, as Pickering emulsion with PFCs cores, PFH and PFP. These emulsions form a unique raspberry‐like structure, with PDA NBs positioned at the droplet interface, with their hydrophobic cavities facing the PFC phase. This morphology is distinct from previously reported PFC emulsions or nanomaterials with conventional PDA shells, mesoporous coatings, or film layers.^[^ ^6^, ^26^, ^27^, ^28^ ^]^ These novel hybrid multiparticle‐armored emulsions combine the droplet stabilizing capability and photothermal conversion capacity of PDA NBs and the thermal responsiveness (phase transition capability) and ultrasound backscattering properties of PFCs making these systems multistimuli‐responsive, phase‐transforming materials.

Our work also pioneers the use of small‐angle and ultra‐small‐angle neutron scattering (SANS and USANS) with contrast variation to characterize these hybrid emulsions. These advanced techniques provide critical insights into the structural and responsive behavior of the PDA NB‐stabilized multiparticle emulsions, which are both thermally and photothermally responsive. SANS and USANS are ideal for exploring material structures at the micro‐ and nanoscale (combined length scale of 1 nm to 10 µm).^[^ ^29^, ^30^ ^]^ The critical advantage of SANS and USANS lies in the ability to match out specific components of the system by adjusting the scattering length density (SLD) of the dispersing medium using different ratios of water (H_2_O) and deuterium oxide (D_2_O). In this case, the PDA NB shell, PFC core, or ensuing microbubbles (upon activation) can be matched out using specific D_2_O–H_2_O mixtures to study the structure or quantity of the other components. By integrating these powerful techniques with conventional characterization methods, our findings highlight the multistimuli‐responsive, phase‐transforming properties of these novel emulsions, combining the stabilizing and photothermal conversion capabilities of PDA NBs with the phase transition and ultrasound backscattering characteristics of PFCs. This comprehensive analysis, integrating SANS and USANS with conventional characterization methods, advances the understanding of structural transformations and phase change kinetics under heat and photothermal activation. These insights are expected to contribute to the development of innovative biomedical materials and enhance their potential for clinical translation in imaging and therapeutic applications.

## 2 Results and Discussion

### 2.1 Fabrication of PDA Bowls and Pickering Emulsions

**Figure 1:**
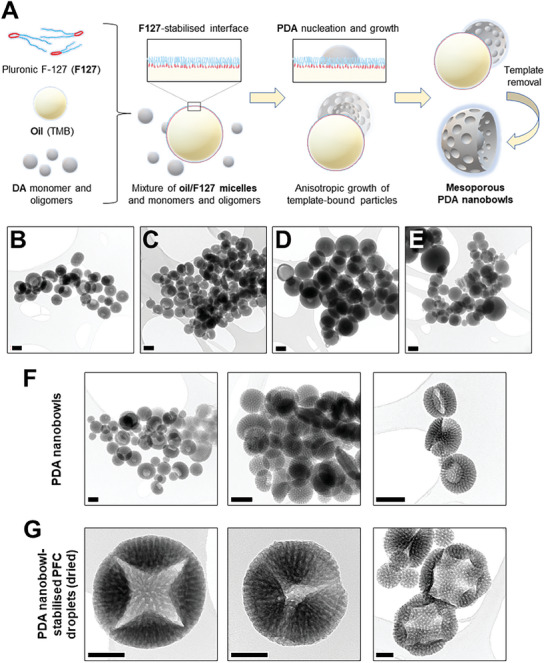
A) Schematic diagram of the PDA NBs fabrication process.^[^ ^9^ ^]^ TEM images of PDA particles from the optimization studies [B) Formulation 1; C) Formulation 2; D) Formulation 3; and E) Formulation 4], F) optimal PDA NB formulation and G) dried remnants of NB‐H emulsion showing PDA NB shell. Scale bars correspond to 200 nm (B–F) and 100 nm (G). Formulation and fabrication parameters and characteristics of the different PDA particle formulations are available in Tables 1 and 2.

Different PDA NBs were fabricated using different process parameters (see Experimental Section and **Table** **1** for the details), based on previously reported methods.^[^ ^17^, ^31^ ^]^ The idealized formation mechanism of mesoporous PDA NBs is shown in **Figure** **1**A.^[^ ^9^ ^]^ With optimized parameters and components, dopamine monomers and oligomers can adsorb onto the soft template interface (1,3,5‐trimethylenzene (TMB) nanodroplets stabilized by Pluronic F‐127), followed by anisotropic particle growth, resulting in the formation of bowl‐shaped nanostructures with mesopores.

**Table 1:** Fabrication process parameters for different PDA NB formulations.

Formulation	DA [g]	F127 [g]	TMB [µL]	Ethanol: Water		NH_3_ [mL]^*^	Stirring time [h]
				ratio	volume [mL]		
1	0.15	0.1	200	5:5	10	0.375	2
2	0.15	0.1	300	5:5	10	0.375	2
3	0.15	0.1	200	8:2	10	0.375	2
4	0.15	0.1	200	5:5	10	0.375	24

* Components: DA – dopamine hydrochloride; F127 – Pluronic F‐127; TMB – 1,3,5‐timethylbenzene; NH_3_ – ammonia solution (28%).

Results of the optimization for PDA NB fabrication are shown in Figure 1B–E and **Table** **2**, Figure S1 and Table S1, Supporting Information. All tested parameters yielded particles with submicron diameters and highly negative ζ‐potential (lower than −30 mV), indicating good colloidal stability. Hydrodynamic diameters from DLS are often larger than the “real” particle sizes observable in TEM, as the former typically overestimates particle size, especially for multimodal or wide particle distributions (i.e., hydrodynamic size represents the size of an ideal sphere with the same diffusive motion as the sample in its environment). Nevertheless, DLS is a quick and reliable particle sizing technique for dilute dispersion, ideal for screening and optimizing PDA NBs. Formulation 1 is based on our previous work,^[^ ^31^ ^]^ which yielded PDA NBs with a monomodal size distribution and a reasonable polydispersity index (PDI = 0.24). Formulation 2, having the highest TMB content, produced the particles with largest diameters and with a bimodal size distribution (modal values at ≈300 and < 500 nm, Figure S1 and Table S1, Supporting Information) and a PDI ≈0.27, which is attributable to larger template drop sizes and potential Ostwald ripening or coalescence of the template during fabrication. Formulation 3 has the highest ethanol content, which induced the formation of large polydisperse nanospheres instead of NBs. High ethanol content encourages rapid aggregation of dopamine monomers and oligomers as nanospheres, limiting their adsorption and growth as NBs on the TMB template droplets’ interface.^[^ ^9^ ^]^ Formulation 4 has the longest polymerization time and produced particles with low size polydispersity (≈0.15) and some similar NB structures with Formulation 1; however, this long incubation time also allowed the excess dopamine oligomers to aggregate and contribute to the formation of particles that are neither bowls nor spheres. Due to the small particle size and uniformity of the NB structures produced by Formulation 1, this set of parameters was used to fabricate the NBs for Pickering emulsions.

**Table 2:** DLS hydrodynamic diameter (Z‐average), TEM‐measured diameter, surface charge, and observed particle morphology of polydopamine nanoparticles produced using different fabrication process parameters.

Formulation	Diameter, DLS^*^ [nm]	Diameter, TEM^**^ [nm]	PDI^*^	ζ‐potential^*^ [mV]	Morphology^***^
1	322.2 ± 48.3	275.6 ± 43.0	0.24 ± 0.01	−36.7 ± 1.8	NBs
2	315.0 ± 122.8	227.9 ± 30.9	0.27 ± 0.02	−31.8 ± 1.1	NBs
3	425.8 ± 36.0	341.9 ± 36.4	0.44 ± 0.04	−39.0 ± 0.6	NSs + NBs
4	359.3 ± 126.1	252.6 ± 75.2	0.15 ± 0.02	−33.7 ± 1.2	NBs + Agg

* Average of modal or peak values in DLS number‐weighted size distribution plots presented as mean ± SD from three different sample batches (n ≥ 3). The complete measured size parameters are available in Table S1, Supporting Information.

** TEM diameters presented as mean ± SD from at least 60 particle measurements.

*** Sample morphologies: NBs = nanobowls; NSs = nanospheres; Agg = aggregates or clusters.

Pickering emulsions are emulsions that are stabilized by particles at the interface. One of our recent works demonstrated the possibility of utilizing PDA NBs as a surfactant‐free stabilizer for emulsions.^[^ ^17^ ^]^ In the current work, PFCs, specifically PFH and PFP, were chosen as the oil core due to their applications in oxygen delivery, biomedical imaging, drug delivery, and theranostics.^[^ ^6^, ^27^, ^32^, ^33^ ^]^ Pickering emulsions of PDA NBs with PFH (NB‐H) and PFP cores (NB‐P) were prepared via a simple sonication method. Due to the large droplet sizes, polydispersity, and droplet sedimentation (PFH and PFP are denser than water), conventional dynamic light scattering is challenging. TEM imaging of dried NB‐H confirmed the self‐assembled micron‐ to submicron‐sized structures armored with PDA NBs, which are remnants of the shell of the Pickering emulsions (Figure 1F,G).

### 2.2 Qualitative Observation of Phase Transition of Pickering Emulsion

#### 2.2.1 Thermally Triggered Phase Change of Pickering Emulsion

**Figure 2:**
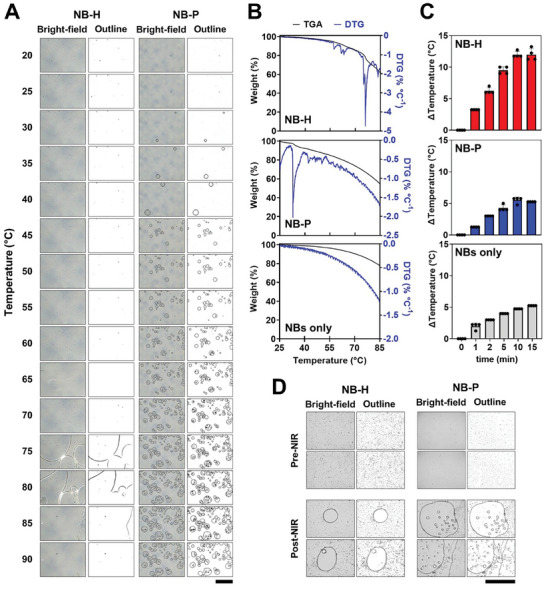
A) Representative optical photomicrographs showing the microbubble production from NB‐H and NB‐P emulsion droplets via heating from 25 to 85 °C. Scale bars = 300 µm. B) TGA and DTG plots of aqueous dispersions of PDA NBs, NB‐H and NB‐P emulsion droplets. C) Normalized temperature change in water, PDA NBs, NB‐H and NB‐P emulsion droplets during NIR illumination (850 nm, 400 mW cm^−2^) over 15 min. Data presented as mean ± SD (n = 4 independent experiments). D) Representative optical photomicrographs showing the microbubble production from NB‐H and NB‐P emulsion droplets photothermal induction over 15 min (850 nm, 400 mW cm^−2^). Scale bars correspond to 300 µm (A,D).

Bubble formation from NB‐H and NB‐P droplets via direct heating was observed using optical microscopy. As shown in **Figure** **2**A, NB‐H did not exhibit bubble formation until ≈75 °C, where larger bubbles violently emerged. NB‐P, on the other hand, exhibited early indications of bubble formation and growth in the temperature range of 20 to 40 °C, followed by a gradual emergence and expansion of numerous stable bubbles that persisted until reaching 90 °C. Imaging was halted at 90 °C to avoid reaching the boiling point of the continuous phase (water).

To support the optical microscopy observation, aqueous dispersion of NB‐H and NB‐P droplets, as well as those of PDA NBs, were subjected to thermogravimetric analysis (TGA, Figure 2B), from which derivative thermogravimetric (DTG) curves were constructed (Figure 2C). TGA and DTG curves of pure PFH and PFP are available in Figure S2, Supporting Information. The decrease in sample mass in the TGA curves can be attributed mainly to the slow evaporation of water (continuous phase) during heating. To accurately identify the phase transition temperatures of the samples, peaks in the DTG curves for all samples were identified. NB‐H exhibited a series of small peaks close to the bulk boiling point of PFH (T_b_ = 56 °C), a wide and strong peak between 70 and 80 °C, and another small peak 80–85 °C. In the case of NB‐P, there is a small peak ≈25 °C, the strongest peak at 30 °C (bulk boiling point of PFP), and multiple smaller peaks between 35 and 60 °C. As expected, no peaks were observed in PDA NBs at the temperature range tested, indicating that water evaporation is the only phenomenon contributing to mass decrease while heating.

The deviation of the observed phase transition temperatures of NB‐H and NB‐P from the bulk boiling points of PFH and PFP, respectively, is an effect of confinement of the PFC cores into small droplets with high degrees of curvature. This confinement results in significantly elevated boiling points of the PFCs, which can be estimated using the Antoine equation (Equation (1)), Laplace pressure (Equation (2)), and the Clausius–Clapeyron equation (Equation (3)) with the following parameters: *P*_1_ and *P*_2_ are the vapour pressures of bulk PFH and PFP droplets, respectively; the Laplace pressure, Δ*P*  =  *P*_2_ − *P*_1_; *T*_1_ and *T*_2_ are the boiling temperatures of bulk PFC and PFC droplets, respectively; *A*, *B*, and *C* are the Antoine parameters for the specific PFC;^[^ ^34^ ^]^ Δ_*vap*_ *H* is the heat of vaporization of the specific PFC; δ is the surface tension of PFC–water interface; *r* is the PFC droplet radius; and *R* is the gas constant (8.314 J mol^−1^ K^−1^).

(1) $$\log\;P_{1}=A-\frac{B}{C+T_{1}}$$

(2) $$P_{2}-\;P_{1}=\frac{2\delta }{r}\;$$

(3) $$\ln\left(\frac{P_{2}}{P_{1}}\right)=\frac{\Delta_{vap}H}{R}\;\left(\frac{1}{T_{2}}-\frac{1}{T_{1}}\right)$$

To illustrate, consider a PFH droplet with *r* = 1 µm and the following parameters: Δ_*vap*_ *H* = 32.4 kJ mol^−1^ and δ (water–PFH) = 56 mN m^−1^;^[^ ^35^, ^36^ ^]^ the expected boiling point would be 83 °C, versus *T_b_* (bulk PFH) = 60 °C. Meanwhile for a PFP droplet (Δ_*vap*_ *H* = 26.6 kJ mol^−1^ and δ (water–PFH) = 54.5 mN m^−1^)^[^ ^35^, ^37^ ^]^ with *r* = 1 µm, the expected boiling point would be 53 °C, versus *T_b_* (bulk PFP) = 30 °C. Predicted boiling points of PFH and PFP droplets with different droplet sizes and interfacial tensions (dependent on the stabilizer used) are shown in Figure S3, Supporting Information. It should be noted that calculations based on the Clausius–Clapeyron equation have several limitations: 1) It assumes a single droplet size; hence, droplets with polydisperse size distributions will require more complex calculations; and 2) It is inapplicable for samples with boiling points close to or beyond the boiling point of the continuous medium (T_b_, H_2_O = 100 °C) upon confinement (size reduction). Nevertheless, the results of these simple calculations for samples boiling below 100 °C corroborate the observation in optical microscopy and TGA, indicating that confinement in droplets is the primary cause of boiling point increase for these emulsified fluorocarbons. For more information regarding the observed DTG peak between 25 and 30 °C in NB‐P droplets, see discussion in Sections 2.3.2 and 2.3.3.

#### 2.2.2 Photothermal Activation of Pickering Emulsions

PDA NBs have been reported to exhibit excellent photothermal conversion capacity in many of our recent works.^[^ ^4^, ^17^, ^38^ ^]^ Figure 2C shows the temperature increase in PDA NBs, NB‐H and NB‐P emulsion droplets over a 15 min NIR illumination (850 nm, 400 mW cm^−2^) period.

Despite the same concentration of NBs in all the samples, NB‐H droplets have the highest temperature change. This can be an effect of thermal accumulation and multiple scattering,^[^ ^39^ ^]^ since the particles are tightly packed and orderly arranged at the interface of large droplets. NB‐H and NB‐P droplets were expected to have similar temperature changes, but since PFP has a lower phase transition temperature (T_b_ (bulk) = 30 °C; T_b_ range of NB‐P droplet starts at ≈40 °C) than PFH (T_b_ (bulk) = 56 °C; T_b_ of NB‐H droplets > 90 °C), the thermal energy was used for phase change. NB‐P droplets and PDA NBs had similar temperature elevations, which are about 5–7 °C lower than NB‐H droplets.

Photothermally induced phase transition of NB‐H and NB‐P were observed qualitatively using optical microscopy imaging. Optical photomicrographs of NB‐H and NB‐P droplets before and after 15‐min NIR exposure are shown in Figure 2D. NB‐P droplets, as expected, showed greater extent of bubble production, in comparison to NB‐H. It can also be observed in both samples that droplet sizes have increased after NIR exposure, indicating that photothermal heating causes either droplet coalescence or Ostwald ripening.^[^ ^40^ ^]^ As larger droplets tend to have lower Laplace pressures than smaller droplets, droplet size increase via these processes facilitates easier transition of the droplets into microbubbles.

### 2.3 SANS and USANS

SANS and USANS offer versatile solutions for exploring the structures and properties of colloidal dispersions, particularly for unique sample systems, such as NB‐H and NB‐P dispersion, which are challenging to study using conventional characterization techniques. As detailed in Sections 2.1 and 2.2, while DLS and ELS are reliable in characterizing the pristine PDA NBs, they are not suitable for measuring the sizes and ζ‐potentials of NB‐H and NB‐P. The high density of PFH and PFP (1.64 and 1.60 g mL^−1^, respectively) and the microscale diameters of NB‐H and NB‐P droplets cause rapid sedimentation, which complicates measurements with DLS and ELS as these techniques require stable dispersions during scans. In contrast, Bilby SANS and Kookaburra USANS, when paired with a sample tumbling system and temperature control, provide an optimal environment for studying NB‐H and NB‐P droplets, addressing the sedimentation issues observed in DLS. Additionally, the integration of temperature control and NIR illumination system (see details in Section 4 – Methodology) enables the study of size changes and phase transformation behavior of these materials under thermal or photothermal triggers. Moreover, scanning these samples in their native dispersion states avoids the structural artifacts, induced by harsh preparation methods and sample environments, previously observed with other PFC emulsions.^[^ ^6^, ^27^, ^41^, ^42^ ^]^

Contrast matching with different water–deuterium oxide mixtures further enhances our ability to examine droplet or microbubble structures during thermally and photothermally triggered phase change of these systems. This approach overcomes the limitations of optical and electron microscopy, which often struggle with differences in sample dimensions, thicknesses, and refractive indices, typically allowing only one structure to be imaged at a time (i.e., one magnification only allows imaging of one structure, either the droplets or the bubbles. Overall, SANS and USANS, when used with the described sample environments and contrast variation techniques, provide a powerful toolkit for in situ characterization of NB‐H and NB‐P transformations and responsiveness.

#### 2.3.1 Contrast Matching Conditions

**Figure 3:**
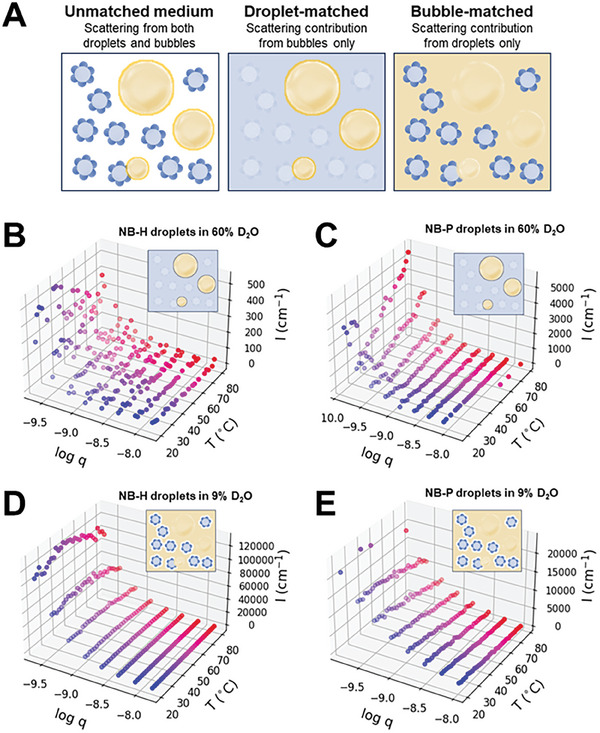
A) Schematic diagram showing the contrast idealized structures of NB‐H and NB‐P emulsion droplets at different contrast matching conditions. 3D plots showing the temperature‐dependence of USANS intensities from NB‐H and NB‐P emulsion droplets in dispersion at different contrast matching conditions: B) NB‐H and C) NB‐P emulsion droplets in PFC droplet‐matched media (60% D_2_O), highlighting scattering from microbubbles; and D) NB‐H and E) NB‐P emulsion droplets in bubble‐matched media (9% D_2_O), highlighting scattering from PFC droplets.

The contrast matching conditions in this work are represented in **Figure** **3**A. To acquire information on the PFC droplets (NB‐H and NB‐P droplets) and microbubbles separately, mixtures with different mass ratios of water (H_2_O) and deuterium oxide (D_2_O) were utilized as dispersing media to match the SLDs of these colloidal species. A 9:91 D_2_O–H_2_O mixture (9% D_2_O, SLD ≈0.00 Å^−2^) was used to match out the neutron scattering from microbubbles (SLD ≈2.6 × 10^−8^ Å^−2^) and highlight the scattering from the liquid PFC droplets (SLD = 3.5 × 10^−6^ Å^−2^).^[^ ^27^, ^41^, ^43^ ^]^ Likewise, scattering from microbubbles was highlighted by matching out scattering from emulsion droplets, using a 60:40 D_2_O–H_2_O mixture (60% D_2_O, SLD = 3.5 × 10^−6^ Å^−2^). Based on our contrast matching experiments with PDA NBs using different D_2_O–H_2_O mixtures as the dispersing media (Figure S4, Supporting Information), these particles have an SLD close to the contrast match point of liquid PFC droplets, that is, at 60% D_2_O, scattering intensities of both PFCs and PDA NBs are either totally matched out or minimized. Furthermore, due to difference in size and concentrations between PFC droplets and PDA NBs, scattering contributions from the latter were expected to be negligible. Hence, scattering from PDA NBs was not accounted for in the subsequent model fitting and analysis.

#### 2.3.2 Phase Change via Direct Heating

**Figure 4:**
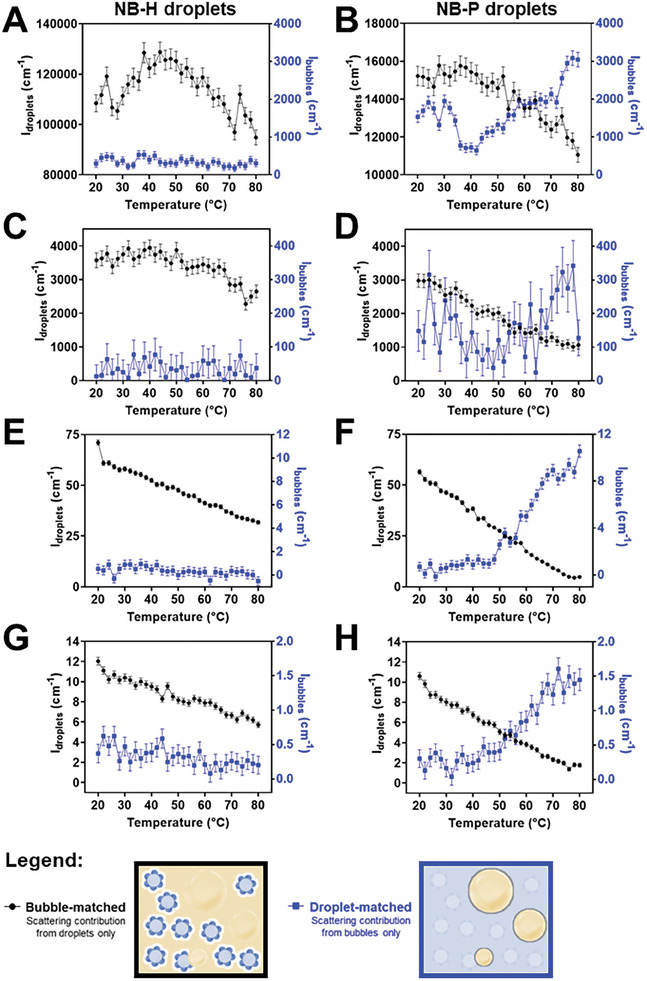
USANS and SANS intensities showing the temperature responsiveness and phase transition of NB‐H and NB‐P emulsion droplets in dispersion at different contrast matching conditions: (blue points) PFC droplet‐matched media (60% D_2_O), highlighting scattering from microbubbles; and (black points) bubble‐matched media (9% D_2_O), highlighting scattering from PFC droplets. Each plot represents scattering intensities at different *q* values/ranges: A) NB‐H and B) NB‐P emulsion droplets at 6.4 × 10^−5^ Å^−1^–7.0 × 10^−5^ Å^−1^ (USANS); C) NB‐H and D) NB‐P emulsion droplets at 1.4 × 10^−4^ Å^−1^ (USANS); E) NB‐H and F) NB‐P emulsion droplets at 2.6 × 10^−3^ Å^−1^ (SANS); G) NB‐H and H) NB‐P emulsion droplets at 3.8 × 10^−3^ Å^−1^ (SANS). Data presented as neutron count rates ± error in neutron counts.

Thermal activation of NB‐H and NB‐P droplets was monitored using SANS and USANS with sample tumbling in the following sequence: 1) full scans to obtain initial scattering patterns of the samples at 20 °C; 2) kinetic scans at different temperatures with less points or shorter scan times than (1); and 3) full scans to obtain the final scattering patterns of the samples after cooling down to 20 °C. USANS intensities from bubbles and PFC droplets (NB‐H and NB‐P droplets) at different *q* values as a function of temperature are presented as 3D plots in Figure 3B–E. SANS patterns of the same samples at different temperatures are presented in Figure S5, Supporting Information. To better represent and understand the kinetics, intensities from these patterns at selected *q* values are extracted and plotted in **Figure** **4**. Different *q* values represent structural changes at different sample length scales. The low‐*q* region in USANS corresponds to length scales of 500 nm to 10 µm, whereas the high‐*q* region in SANS ideally corresponds to length scales of 1 to 500 nm.

Large emulsion droplets of NB‐H (Figure 4A,C) were observed to be resistant to phase change as indicated by a slight droplet signal increase from 20 to 50 °C, followed by a decrease in intensities with no bubble production (stable bubble signal). This behavior aligns with the calculations and experimental results detailed in Section 2.2.1, which suggest a significant elevation in the phase transition temperature of PFH (T_b_ (bulk) = 56 °C) due to confinement within dispersed droplets and increased internal Laplace pressure. The observed increase in droplet signal is attributed to droplet coalescence or Ostwald ripening, as shown by the calculated radii of gyration and radii from Guinier–Porod (Figure S6 and Table S2, Supporting Information). This process leads to the formation of larger droplets that may in part be beyond the USANS *q* range. This hypothesis is also supported by the stable, low signals from bubbles and steadily decreasing droplet signal intensities with increasing temperature in SANS (Figure 4E,G).

On the other hand, NB‐P droplets showed a more pronounced responsiveness to heat with gradual decrease in droplet intensities, associated with significant bubble production starting at 40 °C at low *q* in USANS (Figure 4B) and from 50 °C at intermediate *q* in USANS (Figure 4D) and at high *q* in SANS (Figure 4F,H). Similar to the case of NB‐H, these findings are also consistent with the calculations and experimental results presented in Section 2.2.1, supporting the elevated phase transition temperature of PFP (T_b_ (bulk) = 30 °C) due to increased internal Laplace pressure within the droplets. It is important to note that bubbles are already detectable in the USANS region from 20 to 35 °C (Figure 4B,D, black points), potentially due to the transition of PFP into gas, which stabilized by PDA NBs, during the ultrasonic fabrication step. Sharp interfaces of bubbles and droplet are also indicated by the calculated power laws (≈4.00) from the SANS patterns in Table S3, Supporting Information. NB‐H droplets not showing these power laws in PFC‐droplet matched medium (60% D_2_O) indicate that bubbles with sharp interfaces did not form at the temperatures tested, strongly supporting the kinetics data in Figure 4. Overall, these results reflect the previously reported stabilization (boiling point elevation) of PFC emulsion droplets in aqueous media,^[^ ^27^, ^41^, ^43^, ^44^, ^45^, ^46^, ^47^ ^]^ facilitating the formation of thermally stable emulsions even with smaller PFCs that are gaseous at room temperature.^[^ ^48^, ^49^ ^]^

As shown in Figure S7, Supporting Information, heating to 80 °C followed by cooling to 20 °C caused NB‐H droplet signals to drop with only a very slight increase in bubble signals. This indicates a droplet size increase and partial phase transition, potentially due to the increased droplet sizes as demonstrated in Section 2.2.1. No significant signal change was observed during cooling, indicating that no substantial bubble formation and further droplet structure change eventuated. In the case of NB‐P droplets, bubble and droplet signals were stable during heating at 80 °C. Upon cooling, bubble signals increased and remained stable, which is most likely a result of bubble coalescence or ripening and stabilization by PDA NBs. Droplet signals increased but only reached 75% of the initial intensity. This can be attributed to the coarsening of the remaining droplets that did not undergo phase transition.

During these experiments, certain sample limitations were encountered that influenced the data analysis strategy. While stitching these SANS and USANS patterns is feasible and would enable generation of size distribution plots as demonstrated in our previous works,^[^ ^27^, ^41^ ^]^ the experiments were constrained by insufficient *q* overlap of the data sets. This stemmed from the low sample concentration that had to be maintained (≈2% (v/v)), as exceeding this threshold can induce leaking of the sealed sample cell. At this concentration, phase transition of the droplets can be safely and reliably monitored; however, it is important to note that the USANS data reaches the background at relatively lower *q* values, which restricts stitching with the SANS data. Nevertheless, despite this constraint, it should be emphasized that model fitting can still be performed on these datasets separately.

#### 2.3.3 Phase Change via Photothermal Induction

**Figure 5:**
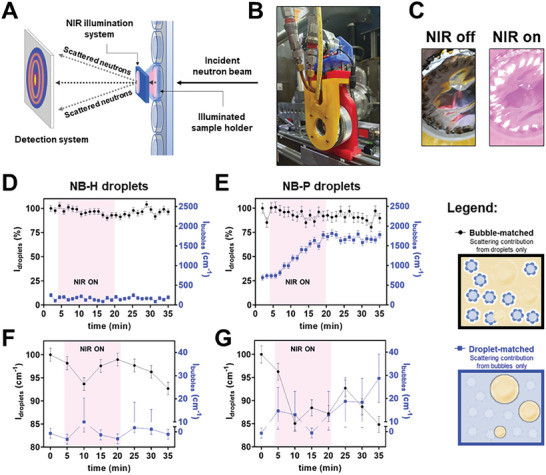
NIR illumination system for the SANS and USANS beamlines: A) schematic diagram of the setup; B) photograph of the assembled NIR illumination system with the sample tumbler, mounted on the Bilby SANS beamline; and C) photographs of the sample window, exposed to the NIR illumination. Plots showing the D,E) USANS and F,G) SANS intensities from D,F) NB‐H and E,G) NB‐P emulsion droplets in dispersion at different contrast matching conditions at different stages of NIR illumination (400 mW cm^−2^) over 15 min. Time ranges highlighted in purple show measurements during NIR illumination (NIR on) period. Data presented as neutron count rates ± error in neutron counts. Different contrast matching conditions are: (blue points) PFC droplet‐matched media (60% D_2_O), highlighting scattering from microbubbles; and (**black points**) bubble‐matched media (9% D_2_O), highlighting scattering from PFC droplets.

**Figure** **5**A–C shows the schematic diagram and photographs of the NIR illumination system, which we used for both Bilby SANS and Kookaburra USANS beamlines (see Figure S8 in the Supporting Information for the mounting ring design and NIR intensity profile). Photothermal activation of NB‐H and NB‐P droplets were monitored using SANS and USANS with sample tumbling in the following sequence: (1) “pre‐NIR” full scan to obtain initial scattering patterns of the samples; (2) 5 min “pre‐NIR” short scan; (3) 15 min NIR illumination; (4) 15‐min “post‐NIR” scan; (5) “post‐NIR” full scan to obtain the final scattering patterns of the samples. It is important to note that Kookaburra USANS and Bilby SANS have different modes of measuring neutron scattering. Bilby SANS can detect the whole SANS *q* range at once using multiple 2D detectors and the resulting patterns can be “time sliced” to monitor the changes in the scattering patterns over time, making it an ideal technique for kinetic measurements. Meanwhile, Kookaburra USANS performs the measurements one *q* value at a time, where points with low intensities (typically at high *q*) may take up to 20 min each to get acceptable statistics. Hence, for kinetic studies, USANS scattering intensities were only measured at a single low *q* value (1.0 × 10^−4^ Å^−1^) to obtain enough points with sufficient statistics during NIR illumination.

Figure 5D demonstrates that the USANS intensities of NB‐H droplets in both bubble‐ and droplet‐matched media remained relatively consistent even during NIR illumination. Notably, in the bubble‐matched medium, USANS intensities of NB‐H droplets initially reduced to ≈90% of their original scattering intensity from liquid PFH droplets. Subsequently, following NIR illumination, there was a recovery to levels ranging from 96% to 104% of the original intensity. Meanwhile, in the case of NB‐P droplets (black points in Figure 5E) within the bubble‐matched medium, there was an initial decrease to about 85% of the original intensity during NIR irradiation, followed by a further decrease to ≈79% post‐NIR, and eventually a recovery to ≈90%. In the droplet‐matched medium, USANS intensities from bubbles displayed an almost linear increase in intensity throughout the duration of NIR illumination, followed by a stable and sustained high signal post‐NIR. These observed USANS signal intensity changes support that NB‐H droplets are also more resistant to photothermal activation, compared to NB‐P droplets.

The samples’ SANS signals in Figure 5F,G also reflect the greater resistance of NB‐H droplets to photothermal activation, in comparison to NB‐P droplets, and possibility of photothermally induced droplet coarsening. NB‐P droplets exhibited more significant SANS signal changes than NB‐H droplets in both contrast matching media: greater extent of droplet depletion (black points) and bubble production (blue points). Droplet coarsening is further substantiated by the results of the Guinier–Porod model fitting, shown in Figure S9 and Table S4, Supporting Information. Both samples showed an increase in droplet size after the 15‐min NIR exposure.

It is worth noting that the signal observed for bubble formation in photothermal activation somehow reaches or exceeds the signals in thermal activation (Figure 4). This is particularly striking considering that the former requires significantly shorter NIR exposure time compared to the latter, requiring 1 h of thermal equilibration. This rapid and robust responsiveness alludes to the potential of PDA NB‐stabilized materials as NIR‐triggered biomedical colloidal material, paving the way for practical translational applications, such as in on‐demand quick‐release drug delivery.

### 2.4 NB‐H and NB‐P Droplets as Ultrasound Contrast Agents

Ultrasound imaging is a non‐invasive, real‐time diagnostic technique widely used for its cost‐effectiveness, accessibility, and portability. It can be used alone or alongside other imaging methods for diagnostics or in combination with treatments and therapeutic strategies. Colloids, like emulsion nanodroplets and microbubbles, enhance ultrasound images, providing better contrast for poorly vascularized organs or distinguishing similar tissues. Ultrasound also serves as an effective tracking system for monitoring colloidal materials with precise targeting capabilities. To assess the feasibility and efficacy of utilizing NB‐H and NB‐P emulsions as contrast agents for ultrasound imaging, as one of their potential applications, their ability to enhance contrast was evaluated through in vitro and in vivo testing.

#### 2.4.1 Acoustic Properties in Tissue‐Mimicking Phantoms (In Vitro Model)

**Figure 6:**
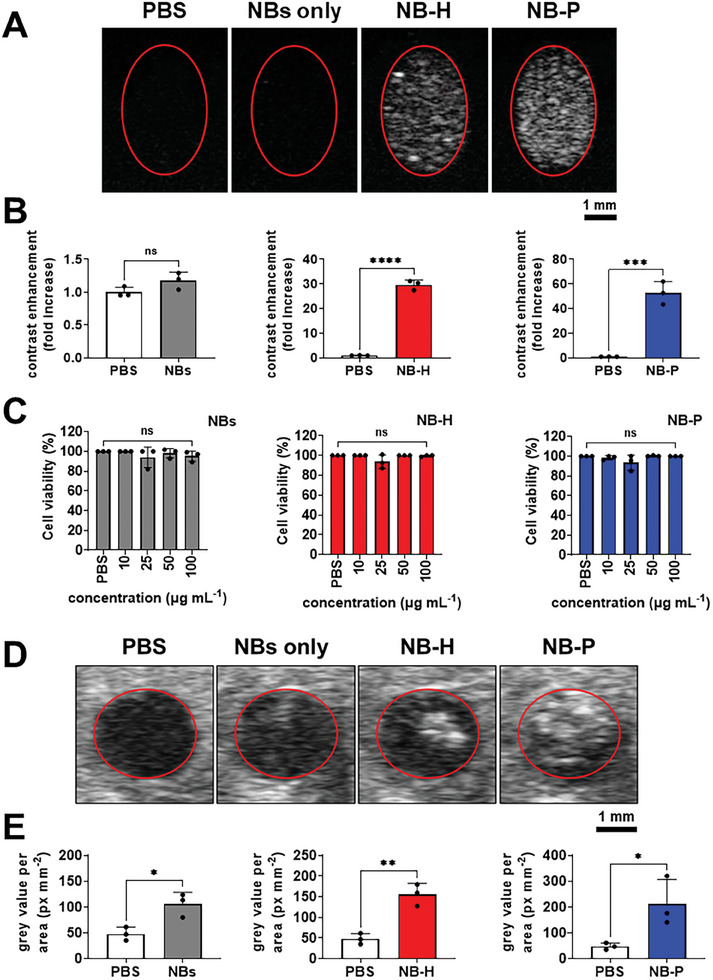
Ultrasound contrast and biocompatibility data of NB‐H and NB‐P droplets. Representative B‐mode ultrasonograms showing the A) wells or hollow regions in tissue‐mimicking phantoms (compartment wall highlighted in red) loaded with PBS, NBs only, NB‐H emulsions, and NB‐P emulsions and B) bar graphs showing their corresponding in vitro ultrasound contrast enhancement. C) MTT cell viability of CHO cells treated with PBS and varying concentrations of NBs, NB‐H emulsions, and NB‐P emulsions (final concentrations of NBs 10, 25, 50, 100 µg mL^−1^). Representative B‐mode ultrasonograms showing the D) inferior vena cava of subject mice injected with PBS, NBs only, NB‐H emulsions, and NB‐P emulsions. Red circles indicate the walls of the inferior vena cava. Scale bars correspond to 1 mm (A,D). E) Bar graphs showing the ultrasound contrast enhancement by the injected samples within the inferior vena cava, represented by the grey value per area. Bar graphs are shown as mean ± SD from three independent experiments (*n* = 3), using Welch's t test (unpaired, two‐tailed) for subpanel B and E, and the Brown‐Forsythe and Welch ANOVA with Dunnett T3 multiple comparisons for C; ns = no significant differences, *p < 0.05, **p < 0.01.

To evaluate the ultrasound contrast enhancement by NB‐H and NB‐P emulsions, freshly prepared dispersions were imaged in hydrogel phantoms (2% agarose) that mimicked the acoustic properties or echogenicity of human tissues. **Figure** **6**A,B demonstrate the strong ultrasound contrast enhancement in brightness (B)‐mode by the NB‐H and NB‐P emulsions, in comparison to NB dispersion (no PFC) and PBS (control).

Ultrasound imaging contrast varies across different colloidal materials and is influenced by several factors, including the properties of the surrounding medium, as well as ultrasound frequency and power settings. Conventional microbubble agents achieve contrast primarily through the high compressibility and low density of their gas cores, as well as factors like bubble size and shell properties (thickness, viscosity, and density).^[^ ^5^, ^50^ ^]^ These bubbles resonate at specific acoustic frequencies and exhibit nonlinear acoustic behavior, enhancing their backscatter signal significantly.^[^ ^51^ ^]^ For liquid droplets and solid colloidal particles, which serve as non‐traditional echo contrast agents, ultrasound contrast relies primarily on simple backscatter, driven by acoustic impedance mismatches with their surroundings. Unlike microbubbles, these materials exhibit weaker contrast since they lack the resonance effects and compressibility of gaseous agents. The acoustic impedance, which determines backscatter intensity, depends on both the material's density (e.g., 1.64 g mL^−1^ for PFH, 1.60 g mL^−1^ for PFP, 0.997 g mL^−1^ for water, and 1.25–1.50 g mL^−1^ for pure, non‐mesoporous PDA^[^ ^9^ ^]^) and the speed of sound through it. Due to their densities, PFC emulsions like NB‐H and NB‐P droplets demonstrate sufficient backscatter to be detected in ultrasonography.

One shared characteristic among colloidal materials is their small size, generally smaller than the imaging ultrasound wavelength, leading to Rayleigh scattering. This results in multidirectional scattering of incident acoustic waves, amplifying the overall acoustic signal. For example, with a 40 MHz ultrasound frequency and an assumed speed of sound in water at 20 °C of 1.48 × 10^3^ m s^−1^, the wavelength of the ultrasound is ≈37 µm. Given that NB‐H and NB‐P emulsions are smaller than this wavelength (as indicated by size measurements in SANS and USANS) with dimensionless wavenumber (*ka*) less than 1 (see calculations in the Supporting Information), they effectively scatter ultrasound in all directions via the Rayleigh scattering effect.^[^ ^52^ ^]^ This multidirectional scattering contributes to the enhanced contrast observed in B‐mode ultrasound imaging.

PFCs, especially low‐boiling point PFP, can also undergo phase transition to form microbubbles either via thermal and photothermal effects as demonstrated in the previous sections of this work or via acoustic droplet vaporization (typically via high‐intensity focused ultrasound),^[^ ^53^ ^]^ which can contribute to the observed ultrasound signals. To compare acoustic signal intensities arising from the different samples and track the origin (bubbles or liquid droplets) of these signals, we imaged PDA NBs, NB‐H, and NB‐P droplets in agarose phantoms at room temperature using both B‐mode and nonlinear contrast (NLC) mode at ≈1.42 MPa and mechanical index (MI) of 0.24. We have also imaged phantoms with just the dispersing medium (water) as vehicle control, and a freshly shaken PDA NB dispersion (to dissolve gases in the container headspace), serving as the gas‐containing control, as well as PDA NB‐stabilized perfluoro‐15‐crown‐5‐ether (PFCE, T_b_ = 146 °C) emulsions (NB‐CE), which is the liquid PFC control as these PFCs are thermally stable and would not undergo ultrasound‐induced phase change or acoustic droplet vaporization (ADV).

As shown in Figure S10, Supporting Information, all PDA NB‐stabilized PFC emulsions, as well as the freshly shaken PDA NB dispersion, displayed strong visual contrast in both B‐mode and NLC mode, indicating that B‐mode and NLC mode can detect both liquid emulsion droplets and bubbles. Since there are no notable differences or trends between the liquid PFC control (NB‐CE) and the samples of interest (NB‐H and NB‐P droplets), attributing the observed acoustic signals specifically to gas bubbles from thermal or acoustic droplet vaporization, particularly in NB‐P droplets, remains challenging. Interestingly, when B‐mode was applied at the highest fixed acoustic power (≈4.48 MPa, MI 0.77), all PDA NB‐stabilized PFC emulsions underwent immediate sedimentation to the bottom of the phantoms, driven by acoustic radiation force from the transducer (Figure S11, Supporting Information). Notably, NB‐P droplets exhibited ADV within seconds of exposure. These findings indicate that the acoustic signals observed in NB‐H droplets are attributed solely to the liquid PFP core, while those from NB‐P droplets could arise from both the liquid PFP and gas bubbles generated through thermally triggered phase change and ADV. Overall, the uncertainty surrounding the exact contributions of liquid and gaseous PFC states to contrast enhancement highlights the need for more detailed studies to delineate the precise activation conditions of PFC droplets under varying ultrasound intensities, mechanical index, and physiological environments.

#### 2.4.2 In Vitro Biocompatibility

Prior to ultrasound imaging in live animals, the biocompatibility of NB‐H and NB‐P droplets at different concentrations was first demonstrated using MTT cell viability assays using Chinese hamster ovarian (CHO) cells as a model for mammalian cells. The results in Figure 6C revealed no significant alteration in the viability of CHO cells treated with NB‐H and NB‐P droplets with PDA NB contents of 10–1000 µg mL^−1^. Interestingly, PDA NBs with concentrations above 100 µg mL^−1^ effected reduced cell viability. It is worth mentioning that the high concentrations tested in this study are at intentionally elevated, suprapharmacological doses (10 to 100 times higher than typically administered doses). It is crucial to emphasize that, in a therapeutic context, upon injection into the bloodstream, these materials undergo immediate dilution, leading to lower systemic concentrations. These results highlight that the PDA NBs, NB‐H and NB‐P droplets are indeed biocompatible with mammalian cells and are promising candidates to be developed as multifunctional colloidal materials for biomedical applications.

#### 2.4.3 Ultrasound Contrast Enhancement in Intravascular Imaging (In Vivo Model)

To evaluate the ultrasound contrast enhancement by the PMBs in a biologically relevant system, sample dispersions were injected into the femoral vein of mice and ultrasonograms of the inferior vena cava were acquired. As shown in Figure 6D,E, approximately two and fourfold increases in ultrasound contrast were observed in the ultrasonograms of mice inferior vena cava after injections of NB‐H and NB‐P emulsions in PBS, respectively. An intriguing finding is the slight contrast enhancement observed in vivo in ultrasonograms with NBs alone, contrasting with the absence of such enhancement in vitro experiments. This disparity may be attributed to the empty cavities within free PDA NBs, acting as nucleation sites for dissolved gases in the blood, thereby facilitating the formation of bubbles capable of effectively backscattering ultrasound.

In these in vivo experiments, we ensured that the injected emulsions were in the liquid phase with no pre‐existing gas bubbles prior to administration. While we observed significant contrast enhancement on ultrasound imaging, we acknowledge that we cannot directly confirm whether gas bubbles were generated from PFC vaporization post‐injection, particularly given that one of the PFC components, PFP, has a very low boiling point. The acoustic signals from NB‐P droplets likely result from backscatter from both the liquid droplets and gaseous PFP bubbles. This is further supported by the accumulation of signals visible at the top of the images, indicating the presence of buoyant bubbles, as NB‐P droplets are already in a superheated state at the body temperature of live mice. As a result, this limitation prevents us from conclusively attributing the observed contrast solely to the liquid phase of the emulsions. Future studies are needed to directly monitor phase transitions in vivo to fully elucidate the mechanisms of contrast enhancement.

Compared to the results of the ultrasonography in tissue‐mimicking phantoms, the observed contrast enhancement by NB‐H and NB‐P emulsions in vivo was significantly lower by orders of magnitude. This primarily stems from dilution of the samples within the bloodstream. Additionally, while simple tissue‐mimicking phantoms exhibited minimal non‐sample scattering, in vivo conditions presented challenges such as the attenuation of the incident ultrasound field due to biological scattering from various layers of skin, fat, muscle, liver, and intestines (and its contents), as well as blood. Despite these challenges, NB‐H and NB‐P emulsions exhibit robust ultrasound backscattering properties and remarkable stability under the potentially destabilizing conditions of vascular circulation. This suggests their potential application as effective ultrasound contrast agents and trackable materials for broader uses in photothermal therapies and theranostics.

## 3 Conclusion

In conclusion, this study presents a comprehensive exploration of the fabrication and phase transition behavior of PDA NB‐stabilized Pickering emulsions, demonstrating their performance and potential applications in biomedical contexts. Leveraging these NBs as Pickering stabilizers in emulsions, particularly with PFH and PFP acting as phase‐changing cores, we have added a new dimension to the versatility of PDA‐based colloids.

Our use of conventional methodologies, complemented by advanced SANS and USANS techniques, represent a significant methodological progress in characterizing these complex colloidal systems. The application of contrast matching conditions enabled a focused and detailed analysis of specific components, unveiling the structural intricacies and phase change kinetics of PDA NB‐stabilized emulsions. Despite some experimental constraints, our findings provide valuable insight into the resistance of NB‐H droplets to phase change and the responsiveness of NB‐P droplets to both thermal and photothermal activation. These results highlight the potential for precise adjustments in the responsiveness and stability of PDA NB‐stabilized emulsions, offering a tailored solution for diverse applications with varying requirements for such properties. The demonstrated efficiency of PDA NBs as photothermal converters further highlights their influence on the phase transition dynamics of emulsion droplets.

The findings bridge critical gaps in understanding the capabilities of PDA NBs, positioning them as promising candidates for drug delivery, imaging, and therapeutic interventions. The integration of neutron scattering techniques enriches our comprehension of the structural transformations within these colloidal systems, setting the stage for further advancements in the realm of multifunctional colloidal materials for biomedicine. These new colloid technologies hold promise for applications as an ultrasound contrast agent for diagnostic imaging. Their strong backscatter properties, resulting from a substantial acoustic impedance mismatch, enhance the visibility of blood vessels and tumors. Modifying these particles with targeting ligands, such as antibodies or peptides, enables selective binding to specific tissues, including tumors and inflamed areas, facilitating targeted imaging of pathological conditions.^[^ ^54^, ^55^ ^]^ These systems can be dual‐functionalized for theranostic applications, combining diagnostic imaging with therapeutic interventions such as image‐guided, photothermal, and sono/photodynamic therapies.^[^ ^56^ ^]^ This approach allows precise control over drug release or therapeutic heat delivery to targeted areas. The responsiveness of these emulsions to stimuli, including heat, near‐infrared light, ultrasound, and magnetic fields, enables on‐demand, localized drug/gene delivery to tumors and cardiovascular lesions.^[^ ^54^, ^57^ ^]^ In cardiovascular imaging, these emulsions can assess blood flow and tissue perfusion in organs such as the heart, liver, and kidneys, aiding in the diagnosis of conditions like ischemia, liver cirrhosis, and other vascular disorders.^[^ ^58^, ^59^ ^]^ Additionally, their high acoustic backscatter makes them promising candidates for sonothrombolysis, enhancing clot visualization and improving the efficacy of ultrasound‐mediated thrombus disruption therapies. Compared to traditional microbubbles, these agents demonstrate enhanced stability in blood flow, increasing their persistence and therapeutic activity. Beyond ultrasound, the incorporation of PFCs also enables imaging using Fluorine‐19 MRI, expanding the versatility of these systems.^[^ ^60^, ^61^ ^]^ We anticipate that these advancements will lead to the development of versatile and personalized treatment strategies for the future.

Ultimately, the knowledge generated from this study can be leveraged to develop innovative multifunctional colloids and biomedical materials, advancing healthcare and therapeutic interventions. Our findings enhance the understanding of PDA NBs and demonstrate their potential for practical application across various biomedical fields, paving the way for personalized and targeted treatment strategies. These advancements represent a significant step forward in the evolution of advanced nanomaterials, contributing to the ongoing progress in theranostics and modern healthcare.

## 4 Experimental Section

### Materials

All chemicals and reagents used in this work were used as received with no further processing unless otherwise specified: PFH (FluoroChem), PFP (Synquest Laboratories), dopamine hydrochloride (Sigma‐Aldrich), 1,3,5‐trimethylbenzene (TMB, 98%, Sigma‐Aldrich), Pluronic F‐127 (Sigma‐Aldrich), ammonia solution (Ajax Finechem Pty.), ethanol (96%, Univa), 3‐(4,5‐dimethylthiazol‐2‐yl)‐2,5‐diphenyltetrazolium bromide (MTT, Invitrogen), and dimethyl sulfoxide (DMSO, Sigma‐Aldrich). Cell culture media and components were supplied by ThermoFisher Scientific: Dulbecco's Modified Eagle Medium (DMEM, high glucose, Gibco) was either used without further processing (FBS‐free medium) or supplemented with 1% *L*‐glutamine, 1% streptomycin/penicillin, and foetal bovine serum (FBS) for cell culture.

The C57BL/6 mice were provided by Alfred Medical Research and Education Precinct (AMREP) Animal Services, under the Alfred Plus Alliance Animal Ethics Committee No. E/1967/2019/B.

### PDA NB fabrication

An emulsion‐induced interfacial anisotropic assembly method was adopted to synthesize PDA bowls.^[^ ^9^, ^31^ ^]^ Fabrication process parameters were In brief, 1.5% (w/v) dopamine hydrochloride and 1.0% (w/v) Pluronic F127 (block copolymer) were dissolved in a water‒ethanol mixture with a total volume of 10 mL. Next, 2.0% (v/v) TMB (oil) was added under stirring, followed by ultrasonication for 2 min to form an emulsion. In the emulsion system, 3.75% (v/v) of ammonia (NH_3_, 28%) solution was added dropwise to achieve an alkaline environment for the reaction to occur. After 2 h (or longer times as noted) of polymerization, the synthesized nanoparticles were centrifuged with water and ethanol three to four times. Subsequently, particles were redispersed in 10 mL of a (1:1) water−ethanol mixture. To increase the stability of the particle dispersion, it was heated in a sealed Teflon‐lined autoclave at 100 °C for 24 h.

We modified the size of the PDA bowls by changing the reaction parameters such as higher concentration of ethanol, prolonged polymerization time, and increasing the usage of TMB, which were summarized in Table 1.

### DLS and ELS

Size and ζ‐potential of PDA particles (1 mg mL^−1^ aqueous dispersion) were measured by dynamic light scattering (DLS) and electrophoretic light scattering (ELS), respectively, using a Malvern Zetasizer (Malvern Panalytical Ltd.).

### Pickering Emulsion Preparation

Emulsions were prepared by mixing 1.0 mg PDA NBs, 35 µL PFH or PFP, and 1.965 mL water (dispersing medium), followed by sonication using a 20 kHz Branson Digital Sonifier SFX 550 (Emerson Electric Co.) for 60 s at 10% amplitude (power output = 3 W) to yield the Pickering emulsion. For samples needed for neutron scattering experiments, deuterium oxide‒water (D_2_O‒H_2_O) mixtures were used as the dispersing media at different contrast matching conditions: 9% D_2_O‒H_2_O to match out bubbles; 60% D_2_O‒H_2_O to match out liquid PFC and minimize scattering from PDA NBs; and 100% D_2_O‒H_2_O as unmatched dispersing medium.

### Thermogravimetric Analysis

TGA was performed on a previously optimized protocol.^[^ ^27^, ^41^ ^]^ Emulsions (5–10 mg) were loaded into standard 100 µL aluminium metal pans with lids and analyzed using a Mettler Toledo TGA‐DSC 1 STARe System with the following parameters: temperature range of 25–85 °C under nitrogen gas flow (30 mL min^−1^), at a heating rate of 3 °C min^−1^.

### TEM

Size and morphology of the PDA NBs were studied using FEI Tecnai T20 TEM at 200 keV. Samples were prepared by drop casting 3.0 µL aliquots of the droplet dispersions onto holey carbon film‐coated, 300 mesh copper grids (EM Solutions), which were then air dried, prior to imaging.

### NIR Setup

The NIR source used for non‐neutron scattering experiments was an OSLON 9 PowerCluster IR, an array of nine OSRAM IR OSLON Black Series LEDs (wavelength = 850 nm), mounted on a heatsink and connected to a DC power source.

### Measurement of Photothermal Heating of PDA/PFC Emulsion Droplets

Samples (100 µL of aqueous dispersions with PDA NBs only,^[^ ^4^, ^27^ ^]^ NB‐H and NB‐P droplets) were placed into the wells of Corning 96‐well, clear polystyrene plates. The plates were covered with lids and then placed 35 mm above the NIR illumination source (luminance = 0.4 W cm^−2^). Temperatures at different time points were measured using a thermocouple. To account for heating from the sample container and the dispersing medium, control experiments were carried out using water with no emulsion droplets.

### Observation of Bubble Formation by Optical Microscopy Imaging

Photomicrographs^[^ ^27^, ^41^ ^]^ of bubble formation induced by an external heat source were obtained using a CCD camera (Flea3, Point Grey, Richmond, BC, Canada) coupled to an Eclipse Ci‐S light microscope (Nikon Instruments, Inc.). Temperature control was achieved using a Peltier temperature stage (Linkam Scientific PE120), coupled to a recirculating water bath, with an accuracy of ± 0.1 °C, at a heating rate of 2.5 °C min^−1^. For NIR illuminated samples, photomicrographs of slides with the samples were obtained before and after exposure to NIR illumination at 0.4 W cm^−2^.

### SANS and USANS

SANS and USANS experiments were performed respectively using the Bilby^[^ ^29^ ^]^ and Kookaburra^[^ ^30^ ^]^ beam‐lines at ACNS ANSTO, Lucas Heights, NSW, Australia, using the methods reported in our previous works.^[^ ^27^, ^41^, ^43^ ^]^ Samples dispersions (1.2 mL) were loaded into 1.5 mL titanium sample cells with quartz windows (40 mm diameter × 1 mm thick), leaving an ≈0.3 mL free volume for gas expansion during heating in the SANS and USANS experiments. The cells were mounted into a tumbling sample holder with an aluminium shroud, where silicon plates cover the sample cells.

The NIR illumination system for the SANS and USANS experiments comes as a removable attachment to the existing Bilby and Kookaburra set‐ups. As shown in Figure 5B and Figure S8, Supporting Information, the illumination system consists of 24 NIR LEDs (wavelength = 860 nm) forming a 44 mm diameter circle, attached to a water‐cooled mounting ring (80 mm diameter). The water‐cooled illumination system is held together by a 3D‐printed holder that adapts to the shape of the ring, providing an unobstructed path for the scattered neutron beam. Each LED is positioned at an angle of 54° from the axis of the mounting ring to direct the NIR beam with mostly uniform intensity (0.4 W cm^−2^) on the sample cell (Figure 5C and Figure S8, Supporting Information).

All neutron scattering measurements were carried out at a set temperature of 20 °C while tumbling. For SANS and USANS measurements, a 17.5 mm borated aluminium aperture and a 12.5 mm cadmium aperture were positioned 9 mm below the centre of the sample cell to avoid the neutron beam from hitting the unfilled part of the cell. SANS measurements were carried out in velocity selector mode with the incident neutron wavelength set at 11 Å. The raw scattering counts were collected on the main detector at a sample–detector distance of 18 m, combined with four curtain detectors at 1.8 and 2.8 m. Data were reduced using the Mantid package,^[^ ^62^ ^]^ resulting in radially averaged intensity data I(*q*) where the scattering vector *q* is defined as:

(4) $$q=\frac{4\pi }{\lambda }\;\sin\left(\frac{\theta }{2}\right)$$

with λ the incident neutron wavelength and θ the scattering angle. Absolute intensity scaling was achieved based on an empty beam transmission measurement, and the simultaneous *q* range was 0.0017–0.2 Å^−1^. For background subtraction, an empty cell measurement was used.

For USANS measurements, Kookaburra utilizes a Bonse‐Hart rocking axis neutron spectrometer, where the monochromator and analyzer consist of two identical arrays of five‐reflection, channel‐cut silicon single crystals, aligned in a non‐dispersive, parallel geometry that produces Bragg reflection conditions. An incident neutron wavelength of 4.74 Å was used. Rocking curve profiles were obtained by rotating the analyzer crystal away from the aligned peak position and measuring the neutron intensity as a function of the scattering vector *q*, point by point. The total *q* range was 0.00003–0.01 Å^−1^, however the highest *q*‐value is often limited by reduced signal‐to‐noise ratio. Full *q* range scans were carried out only on droplet samples dispersed in microbubble‐matched medium (9:91 D_2_O–H_2_O). Kinetic scans, involving scattering measurements at selected *q* values before, during and after NIR illumination were carried out on both droplet‐ and microbubble‐matched dispersing media (61:39 and 9:91 D_2_O–H_2_O, respectively). Data were reduced by using Python scripts in Gumtree,^[^ ^63^ ^]^ based on a standard procedure.

### SANS and USANS Data Analysis

USANS data were fitted to models using SasView software (https://www.sasview.org). A Guinier–Porod model was used to fit the USANS data for samples scanned in 9% D_2_O (microbubble‐matched). Since the Guinier region was beyond the *q* range of the bubbles, data obtained from samples in a 61% D_2_O (emulsion‐matched) were fitted using a power‐law model.

### Ultrasound Imaging

The ultrasound contrast‐enhancing capabilities of PDA NBs, NB‐H, and NB‐P emulsions were evaluated via ultrasound imaging using a Vevo 2100 high‐frequency ultrasound scanner with a 22–55 MHz MS 550D transducer (FUJIFILM VisualSonics, Inc.) with an operating frequency of 40 MHz. Imaging was conducted with a duty cycle of 100% in two imaging modes: brightness mode (B‐mode) at 100% transmit power with free‐field values for peak rarefactional pressure = 4.48 MPa and mechanical index = 0.77; and non‐linear contrast (NLC) mode, which simultaneously provides a B‐mode view and a contrast imaging view at lower transmit power (6–10%), theoretical peak rarefactional pressure (1.42 MPa) and mechanical index (0.24).

### In Vitro Model for Ultrasound Imaging

Ultrasonograms were obtained from tissue‐mimicking phantoms made from 1% agarose hydrogels which were loaded with different concentrations of NB‐H and NB‐P droplets. PDA NBs and PBS were used as controls with three independent experiments (n = 3). B‐mode and NLC mode imaging were performed, with each experiment lasting 1 min. For image analysis, the first 100 frames (5 s ultrasound exposure) from the B‐mode ultrasonograms were analyzed. This approach ensured that the selected frames reflected the system with minimal influence from sample sedimentation (as the system is static) or uncontrolled bubble formation, reducing artifacts and variability in contrast.

### In Vivo Model for Ultrasound Imaging

All animal experiments involving ultrasound imaging were approved by the Alfred Medical Research and Education Precinct Animal Ethics Committee (approval E/8335/2022/B). C57BL/6 mice were injected with a combination of ketamine (100 mg kg^−1^) and xylazine (5 mg kg^−1^) intraperitoneally. Then a 1 cm incision was made between the abdomen and thigh of the mouse. A catheter was inserted intravenously into the exposed femoral vein and secured. The mouse was then placed onto a VisualSonics imaging station (VisualSonics Inc, Canada) in a supine position. The ultrasound probe was positioned over the abdominal area to locate and obtain a clear image of the inferior vena cava (IVC), while taking care to avoid shadow artifacts caused by intestinal contents (see sample ultrasonograms in Figure S12, Supporting Information and Videos S1 and S2, Supporting Information for anatomy annotation). PBS (control) or samples (100 µL containing either PDA NBs, NB‐H or NB‐P droplets) were administered via the catheter Ultrasound images of the IVC using B‐mode and NLC mode (*n* = 3 with multiple frames for each animal) were captured over a 5‐min period. Grey values from B‐mode ultrasonogram frames acquired within the first 30 s of each sample administration were analyzed using ImageJ. This approach ensured that the selected frames reflected the system with minimal influence from sample dilution and clearance over time, reducing variability in observed contrast.

### MTT Assay

Viability of Chinese Hamster ovary (CHO) cells after exposure to NBs, NB‐H, and NB‐P emulsions was evaluated using a standard MTT protocol.^[^ ^64^ ^]^ Briefly, cells (1 × 10^5^ cells per well) were seeded into 96‐well plates in 100 µL volume with cell culture medium (DMEM supplemented with 10% (v/v) FBS, 1% (v/v) penicillin–streptomycin solution and 1% (v/v) L‐glutamine) and incubated for 24 h at humidified conditions (37 °C) with 5% CO_2_ supply using Steri‐cycle CO2 Incubator (Thermo Fischer Scientific, Germany). After 24 h, the medium from each well was replaced with fresh medium containing different concentration of NBs, NB‐H, and NB‐P emulsions (10, 50, 100 µg mL^−1^ NB content), followed by incubation at 37 °C for 24 h (*n* = 3). MTT solution (10 µL, 5 mg mL^−1^) was then added per well, followed by a 4‐h incubation period at 37 °C. After incubation, the medium was removed and replaced with 100 µL DMSO to each well to dissolve formazan crystals, followed by spectrophotometric reading at 570 nm using a microplate reader (BMG Labtech FLUOstar Omega Microplate Reader, Germany). Cell viability was calculated and normalized to 100% using the absorbance of wells with PBS‐treated cells.

### Statistical Analysis

All data were assessed for normality using the Shapiro‐Wilk test. Evaluation of outliers was determined using ROUT (Q = 0.1% for definitive outliers). An F test was used to analyze equality of variance for data sets with two groups, whereas the Brown‐Forsythe test was used for data sets with more than three groups. Data was reported as mean ± standard deviation (SD). The sample size (n) for each statistical analysis is clearly stated in each respective section (n ≥ 3). Statistical methods are as follows: for data sets of two groups with parametric data and equal or unequal variance, statistical analysis was performed using the Welch's t test (two‐tailed); and for data sets of more than two groups, one‐way ANOVA followed by post hoc analysis by Tukey test was used. In the event of unequal variance, the Brown‐Forsythe and Welch ANOVA with Dunnett T3 multiple comparisons was used. Test results were considered statistically significant at values of *p* < 0.05 using GraphPad Prism v9.0.

## Conflict of Interest

The authors declare no conflict of interest.

## Author Contributions

M.L.P.V. and H.L. contributed equally to this work. **Mark Louis P. Vidallon** – Conceptualization, Methodology, Validation, Investigation, Resources, Data Curation, Formal Analysis, Visualization, Funding Acquisition, Writing – Original Draft, Writing – Review & Editing; **Haikun Liu** – Conceptualization, Methodology, Investigation, Data Curation, Formal Analysis, Visualization, Writing – Original Draft, Funding Acquisition, Writing – Review & Editing; **Zhenzhen Lu** – Conceptualization, Methodology, Investigation, Data Curation, Funding Acquisition, Writing – Original Draft; **Shahinur Acter** – Conceptualization, Methodology, Investigation, Data Curation, Funding Acquisition, Writing – Original Draft; **Yuyang Song** – Methodology, Investigation; **Chris Baldwin** – Conceptualization, Methodology, Investigation; **Alexis I. Bishop** – Conceptualization, Methodology, Investigation, Funding Acquisition, Writing – Review & Editing; **Boon Mian Teo** – Conceptualization, Methodology, Funding Acquisition; **Rico F. Tabor**– Conceptualization, Methodology, Resources, Funding Acquisition, Writing – Review & Editing; **Karlheinz Peter**– Resources, Supervision, Project Administration, Data Curation, Funding Acquisition, Writing – Review & Editing; **Liliana de Campo** – Conceptualization, Methodology, Validation, Investigation, Data Curation, Formal Analysis, Visualization, Funding Acquisition, Writing – Original Draft, Writing – Review & Editing; **Xiaowei Wang** – Conceptualization, Methodology, Validation, Investigation, Resources, Supervision, Project Administration, Data Curation, Formal Analysis, Visualization, Funding Acquisition, Writing – Original Draft, Writing – Review & Editing.

## Supporting information



Supporting Information

Supporting Information

Supplemental Video 1

Supplemental Video 2

## Data Availability

The data that support the findings of this study are available from the corresponding author upon reasonable request.
