# Supplementary material for: Polydopamine Nanobowl‐Armoured Perfluorocarbon Emulsions: Tracking Thermal‐ and Photothermal‐Induced Phase Change through Neutron Scattering
Source: Small. 2024 Nov 10;21(2):2406019. doi: 10.1002/smll.202406019 (PMC11735900; doi:10.1002/smll.202406019)
Supplement: Supplementary file 1 — Supporting Information [file SMLL-21-2406019-s003.docx]

**SUPPORTING INFORMATION**

**Polydopamine Nanobowl-Armoured Perfluorocarbon Emulsions: Tracking Thermal- and Photothermal-Induced Phase Change through Neutron Scattering**

Mark Louis P. Vidallon,^1,2,3,4,†,^* Haikun Liu,^1,2,†^ Zhenzhen Lu,^5^ Shahinur Acter,^6^ Yuyang Song,^1,2^ Chris Baldwin,^7^ Boon Mian Teo,^3^ Alexis I. Bishop,^8^ Rico F. Tabor,^3^ Karlheinz Peter,^2,4,9,10^ Liliana de Campo,^7,^* and Xiaowei Wang^1,2,4,10,^*

^1^ Molecular Imaging and Theranostics Laboratory, Baker Heart and Diabetes Institute, 75 Commercial Road, Melbourne, VIC, 3004, Australia

^2^ Baker Department of Cardiometabolic Health, University of Melbourne, Parkville, VIC, 3010, Australia

^3^ School of Chemistry, Monash University, Clayton, VIC, 3800, Australia

^4^ Baker Department of Cardiovascular Research, Translation and Implementation, La Trobe University, Bundoora, VIC, 3086, Australia

^5^ Department of Chemical Engineering, University of Melbourne, Parkville 3010, Australia

^6^ Department of Radiation Oncology and Molecular Sciences, The Johns Hopkins School of Medicine, Johns Hopkins University, 733 N Broadway, Baltimore, MD, 21205, USA

^7^ Australian Nuclear Science and Technology Organization (ANSTO), New Illawarra Rd, Lucas Heights, NSW, 2234, Australia

^8^ School of Physics and Astronomy, Monash University, Clayton, VIC, 3800, Australia

^9^ Atherothrombosis and Vascular Biology Laboratory, Baker Heart and Diabetes Institute, 75 Commercial Road, Melbourne, VIC, 3004, Australia

^10^ School of Translational Medicine, Monash University, Melbourne, VIC, 3004, Australia

^†^ These authors contributed equally to this work.

Correspondence to: Prof. Xiaowei Wang ([xiaowei.wang@unimelb.edu.au](mailto:xiaowei.wang@unimelb.edu.au)) and Dr. Mark Louis P. Vidallon (marklouis.vidallon@unimelb.edu.au); Baker Department of Cardiometabolic Health, University of Melbourne, Parkville, VIC, 3010, Australia; and Dr. Liliana de Campo ([liliana.decampo@ansto.gov.au](mailto:liliana.decampo@ansto.gov.au)); Australian Nuclear Science and Technology Organisation (ANSTO), New Illawarra Rd, Lucas Heights, NSW, 2234, Australia


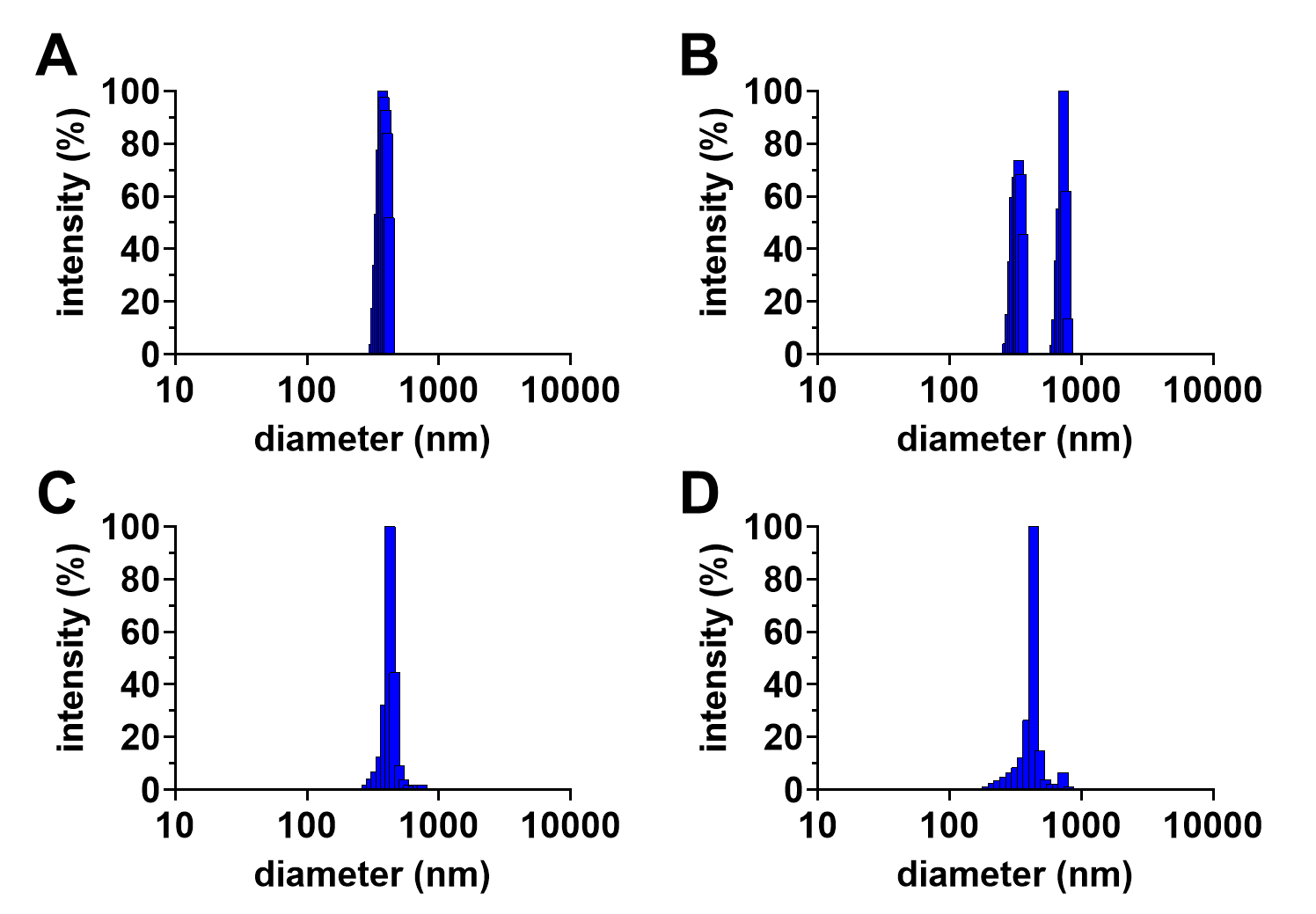


**Figure S1**. Representative intensity-weighted DLS size distribution plots of polydopamine nanoparticles produced using different fabrication process parameters: (**A**) Formulation 1; (**B**) Formulation 2; (**C**) Formulation 3; and (**D**) Formulation 4. Details of the fabrication method and the complete list of parameters are available in Section 4.5 and Table 1, respectively, in the main document.

**Table S1.** DLS hydrodynamic diameters (Z-average) and average modal values from intensity-weighted and number-weighted size distributions of polydopamine nanoparticles produced using different fabrication process parameters.

| **Formulation** | **Diameter, Z-average* (nm)** | **Modal diameter, intensity-weighted** (nm)** | **Modal diameter, number-weighted** (nm)** |
| --- | --- | --- | --- |
| 1 | 456.5 ± 40.0 | 343.9 ± 25.5 | 322.2 ± 48.3 |
| 2 | 694.6 ± 54.5 | 292.7 ± 131.1  915.3 ± 626.8*** | 315.0 ± 122.8***  581.3 ± 139.3 |
| 3 | 558.2 ± 88.5 | 622.8 ± 219.5 | 425.8 ± 36.0 |
| 4 | 441.1 ± 4.1 | 407.6 ± 13.0 | 359.3 ± 126.1 |

*Data presented as mean ± SD from at least three different sample batches (n ≥ 3).

**Data presented as mean ± SD of modal or peak values from at least three different sample batches (n ≥ 3).

***The major peak in the bimodal size distribution. Note that intensity-weighted size distributions are biased toward larger particles, which scatter light more effectively than smaller particles (see Figure S1B for an example). In contrast, number-weighted size distribution reallocates emphasis to the population with the higher calculated particle count, as it is based on particle abundance rather than scattering intensity.


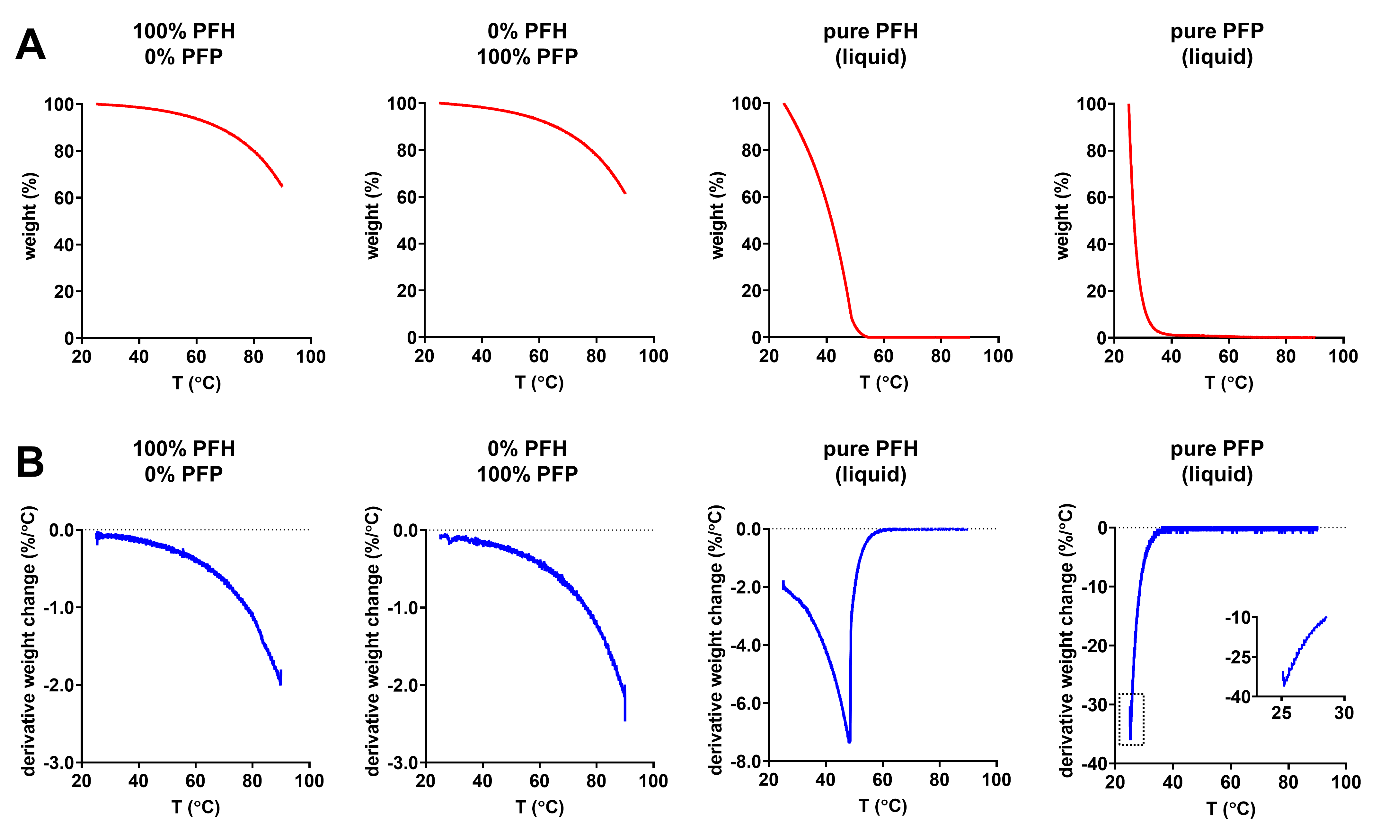


**Figure S2**. Thermogravimetric (*top*) and derivative thermogravimetric curves (*bottom*) of pure PFH and PFP.


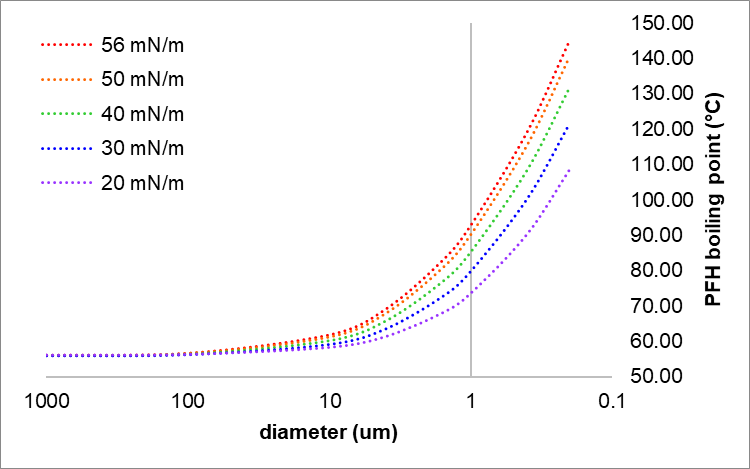

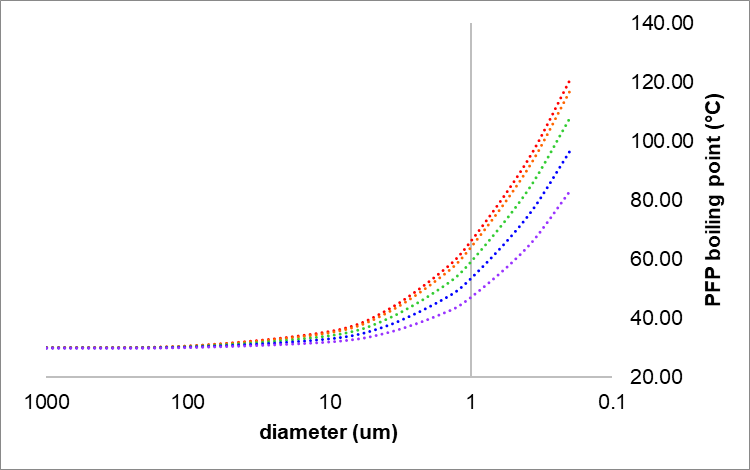


**Figure S3.** Predicted boiling points of (**top**) PFH and (**bottom**) PFP droplets with different droplet diameters and interfacial tensions.

**
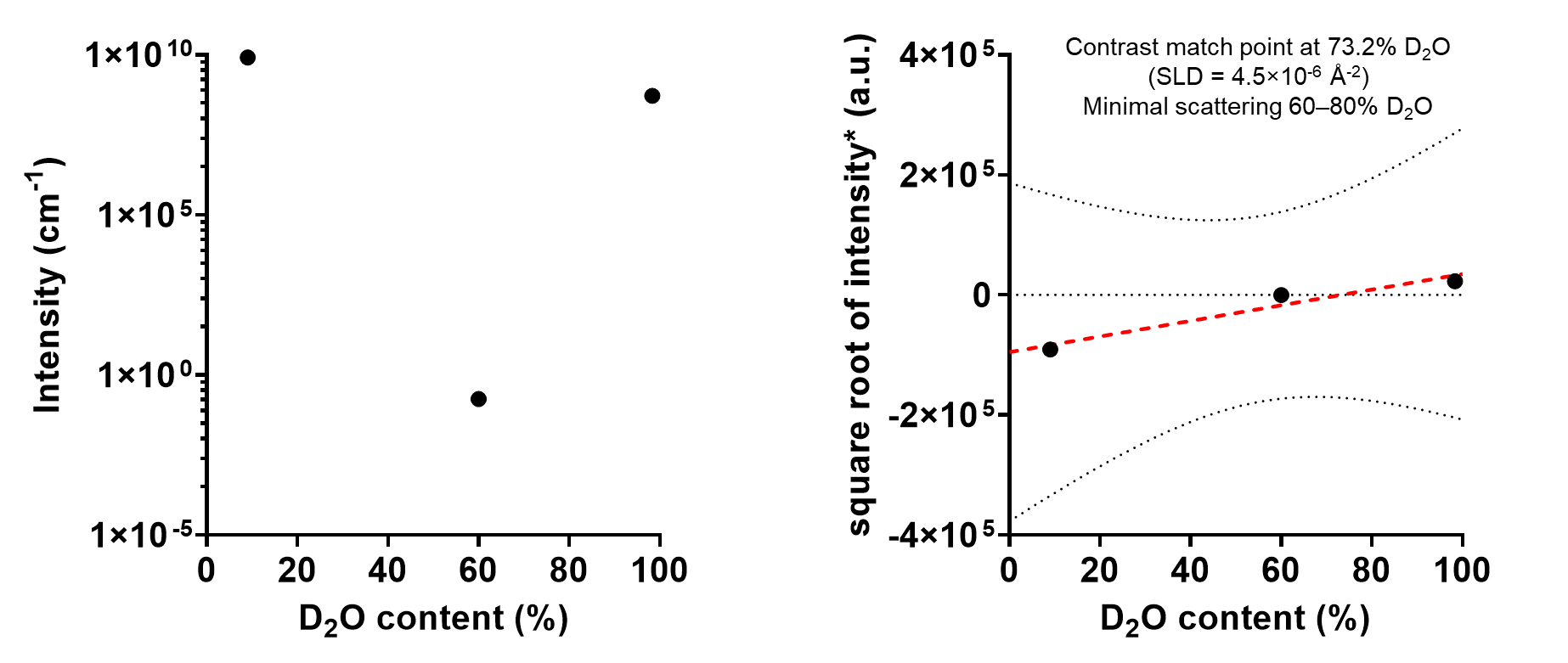
**

**Figure S4.** Plots showing the results of neutron contrast matching experiments with PDA nanobowls: (**A**) scattering intensity data and (**B**) linearized scattering data from sample dispersion in different D_2_O‒H_2_O mixtures, showing the interpolated SLD of PDA nanobowls close to 4.5×10^-6^ Å^-2^.


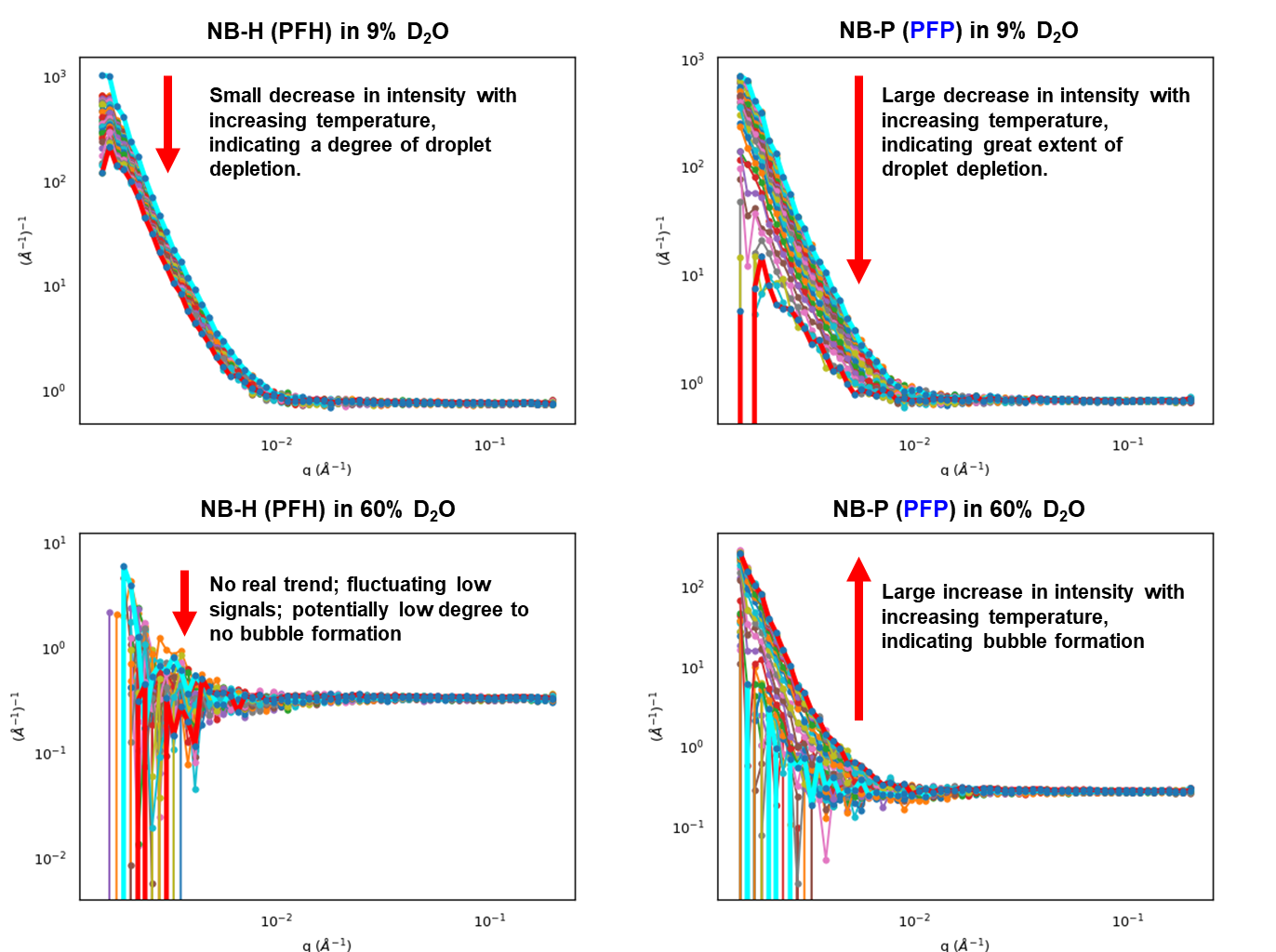


**Figure S5.** SANS patterns from NB-H (PFH) and NB-P (PFP) in 9% and 60% D_2_O at different temperatures (20°C in cyan to 80°C in red).

**
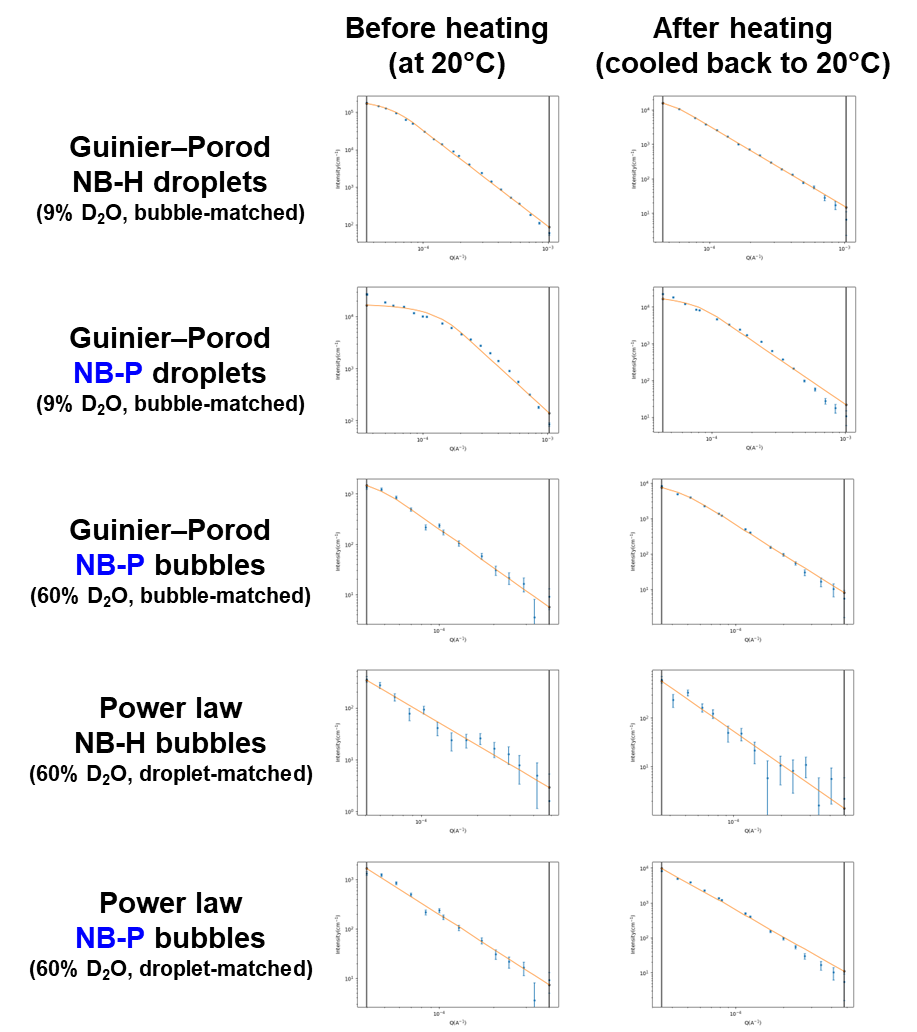
**

**Figure S6.** Plots showing the Guinier–Porod and power law fits of USANS data from NB-H (PFH) and NB-P (PFP) in 9% and 60% D_2_O, before and after heating. Data for NB-H bubbles were not fitted with the Guinier–Porod model (only power law) due to the noisy signals and weak scattering intensities, close to background.

**Table S2.** Fitting parameters from the Guinier–Porod model and power law fitting of NB-H and NB-P droplet dispersions in bubble-matched and droplet-matched media before and after heating. Parameters obtained correspond to model fitting in Figure S6.

| **Sample** | **Parameter** | **Before heating*** | **After heating*** |
| --- | --- | --- | --- |
| NB-H droplets  (9% D_2_O, Guinier–Porod**) | Rg (nm) | 2756 (31) | 3035 (96) |
|  | Porod | 3.56 (0.01) | 3.33 (0.02) |
|  | χ2 | 8.77 | 1.04 |
| NB-P droplets  (9% D_2_O, Guinier–Porod**) | Rg (nm) | 1048 (14) | 1991 (36) |
|  | Porod | 3.20 (0.02) | 3.48 (0.02) |
|  | χ2 | 43.61 | 28.68 |
| NB-P bubbles  (60% D_2_O, Guinier–Porod**) | Rg (nm) | 3601 (262) | 3897 (117) |
|  | Porod | 3.55 (0.10) | 3.79 (0.04) |
|  | χ2 | 2.24 | 2.26 |
| NB-H bubbles  (60% D_2_O, Power law) | Power law*** | 3.11 (0.13) | 3.28 (0.15) |
|  | χ2 | 0.97 | 1.21 |
| NB-P bubbles  (60% D_2_O, Power law) | Power law*** | 3.35 (0.06) | 3.56 (0.02) |
|  | χ2 | 3.31 | 7.00 |

*Values in parentheses are the SasView error values for the fitted parameters.

**Fixed parameter (Guinier–Porod model): s = 0 (3D globular objects, such as spheres).

***Note that the scattering intensity from bubbles (samples at 60% D_2_O) are close to background; parameters from the model fitting might not be reliable and lack meaningful interpretation.

**Table S3.** Power law of SANS patterns from NB-H (PFH) and NB-P (PFP) in 9% and 60% D_2_O at different temperatures (20–80°C, then back to 20°C).

| **T (°C)** | **Power law*** | | | |
| --- | --- | --- | --- | --- |
|  | **NB-H (PFH)**  **in 9% D_2_O** | **NB-H (PFH)**  **in 60% D_2_O** | **NB-P (PFP)**  **in 9% D_2_O** | **NB-P (PFP)**  **in 60% D_2_O** |
| 20 | 4.82 (0.10) | 2.03 (0.40) | 4.49 (0.04) | 4.38 (1.50) |
| 22 | 4.75 (0.10) | 2.94 (0.36) | 4.37 (0.04) | -1.06 (1.52) |
| 24 | 4.77 (0.12) | 1.55 (0.28) | 4.48 (0.04) | -5.26 (1.16) |
| 26 | 4.74 (0.12) | 0.83 (0.36) | 4.41 (0.04) | -15.80 (7.63) |
| 28 | 4.80 (0.13) | -5.02 (0.54) | 4.45 (0.04) | -0.58 (31.12) |
| 30 | 4.81 (0.13) | -11.83 (4.93) | 4.48 (0.04) | -0.43 (1.02) |
| 32 | 4.76 (0.13) | -0.88 (0.06) | 4.41 (0.04) | -0.65 (0.58) |
| 34 | 4.81 (0.13) | -0.48 (1.20) | 4.45 (0.05) | 0.44 (0.56) |
| 36 | 4.70 (0.13) | -0.83 (0.61) | 4.46 (0.05) | 0.20 (0.29) |
| 38 | 4.79 (0.14) | -3.58 (1.46) | 4.49 (0.05) | -1.15 (0.28) |
| 40 | 4.66 (0.14) | -0.89 (1.81) | 4.54 (0.05) | -3.89 (1.85) |
| 42 | 4.69 (0.14) | -4.52 (2.42) | 4.51 (0.05) | 0.69 (3.03) |
| 44 | 4.80 (0.14) | -1.13 (1.28) | 4.45 (0.05) | -0.72 (0.51) |
| 46 | 4.59 (0.14) | -0.39 (0.86) | 4.40 (0.06) | 3.61 (0.54) |
| 48 | 4.75 (0.15) | -1.19 (0.87) | 4.40 (0.06) | 4.84 (0.88) |
| 50 | 4.70 (0.15) | -1.08 (1.00) | 4.45 (0.06) | 4.74 (0.56) |
| 52 | 4.65 (0.15) | -2.11 (1.28) | 4.39 (0.06) | 5.74 (0.54) |
| 54 | 4.64 (0.16) | -1.63 (0.76) | 4.33 (0.07) | 4.87 (0.88) |
| 56 | 4.69 (0.16) | -2.69 (1.71) | 4.33 (0.07) | 4.79 (0.56) |
| 58 | 4.67 (0.17) | -3.70 (2.51) | 4.53 (0.08) | 5.27 (0.54) |
| 60 | 4.58 (0.17) | -1.19 (1.20) | 4.60 (0.09) | 4.90 (0.41) |
| 62 | 4.50 (0.17) | -1.66 (1.20) | 4.56 (0.10) | 5.89 (0.36) |
| 64 | 4.72 (0.18) | -1.02 (0.73) | 4.31 (0.10) | 5.26 (0.29) |
| 66 | 4.60 (0.18) | -1.34 (0.75) | 4.30 (0.11) | 5.13 (0.25) |
| 68 | 4.68 (0.19) | -0.66 (0.69) | 4.32 (0.12) | 4.81 (0.31) |
| 70 | 4.58 (0.19) | -1.06 (0.96) | 4.32 (0.14) | 4.94 (0.21) |
| 72 | 4.59 (0.19) | -0.94 (0.56) | 4.10 (0.16) | 4.94 (0.20) |
| 74 | 4.60 (0.20) | -1.75 (1.00) | 4.43 (0.21) | 4.84 (0.16) |
| 76 | 4.63 (0.21) | -1.65 (1.15) | 4.34 (0.26) | 4.80 (0.16) |
| 78 | 4.61 (0.21) | -1.12 (0.68) | 3.77 (0.19) | 4.80 (0.15) |
| 80 | 4.70 (0.23) | -0.26 (0.50) | 4.14 (0.23) | 4.80 (0.15) |
| 80 | 4.75 (0.23) | -0.79 (0.49) | 4.16 (0.25) | 4.55 (0.15) |
| 80 | 4.58 (0.23) | -0.49 (0.47) | 3.75 (0.19) | 5.04 (0.14) |
| 80 | 4.51 (0.21) | -0.62 (0.50) | 3.81 (0.19) | 4.74 (0.14) |
| 20 | 4.58 (0.19) | -0.57 (0.51) | 4.28 (0.10) | 5.02 (0.08) |

*Values in parentheses are the SasView error values for the fitted parameters.

**Note**: Calculated power laws are generally close to 4, indicating sharp droplet or bubble interfaces. Patterns from samples in 60% D_2_O, NB-H and NB-P until 44°C, are very noisy and close to background; hence, these power law values are grey and lack any meaningful interpretation and should be disregarded.


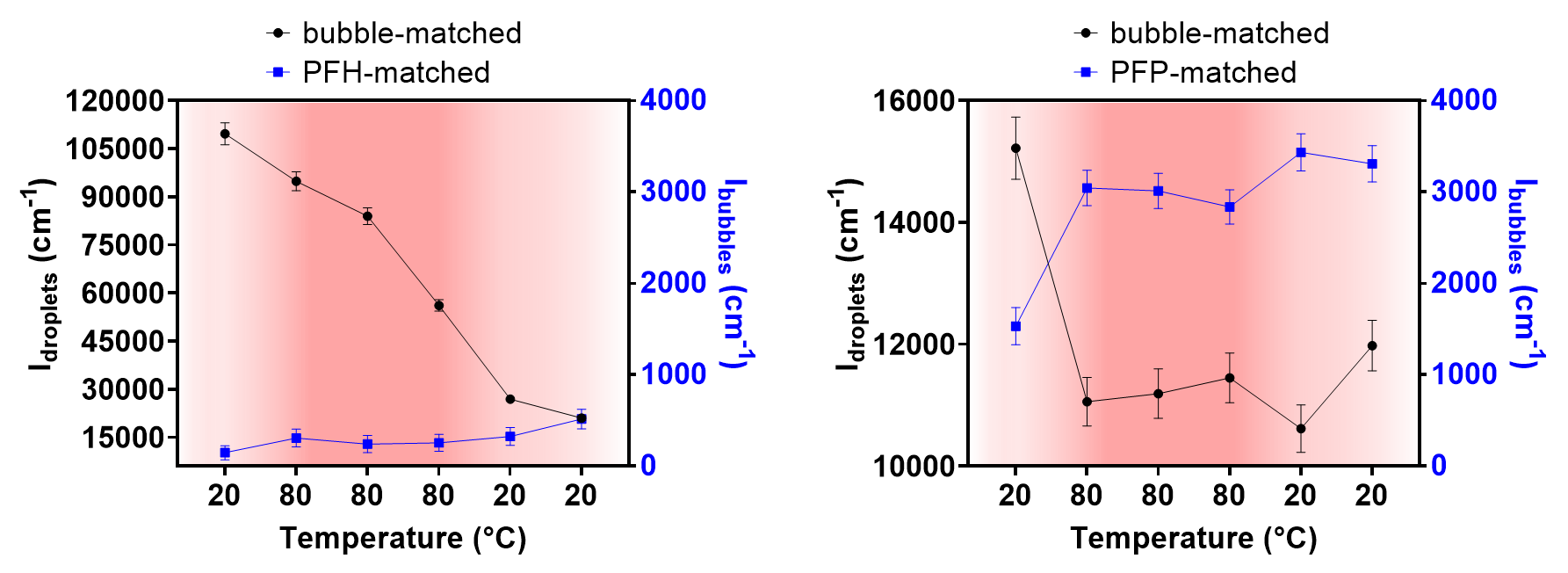


**Figure S7.** USANS signals showing the temperature responsiveness and phase transition of (*left*) NB-H and (*right*) NB-P emulsion droplets in dispersion at different contrast matching conditions: (**blue points**) PFC droplet-matched media (60% D_2_O), highlighting scattering from microbubbles; and (**black points**) bubble-matched media (9% D_2_O), highlighting scattering from PFC droplets. Each plot represents scattering intensities at 6.4×10^-5^ Å^-1^–7.0×10^-5^ Å^-1^. Data presented as neutron count rates ± error in neutron counts.


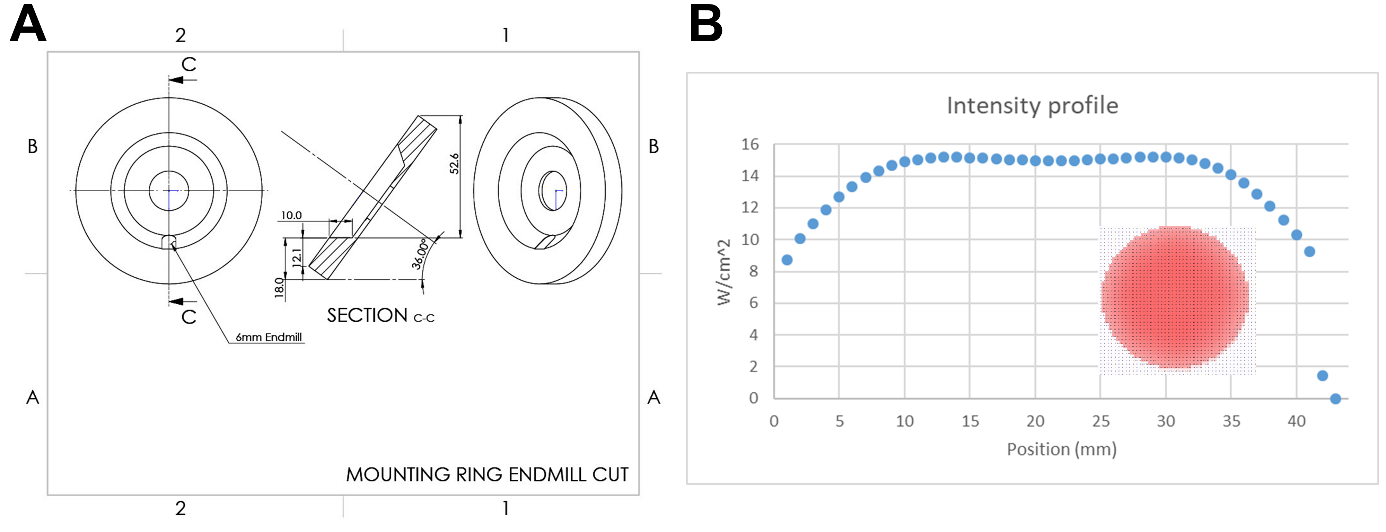


**Figure S8**. (**A**) Schematic diagram showing the dimensions of the mounting ring. (**B**) NIR intensity profile of the NIR illumination system. Inset in B shows the NIR intensity profile on a 44-mm diameter surface.

**
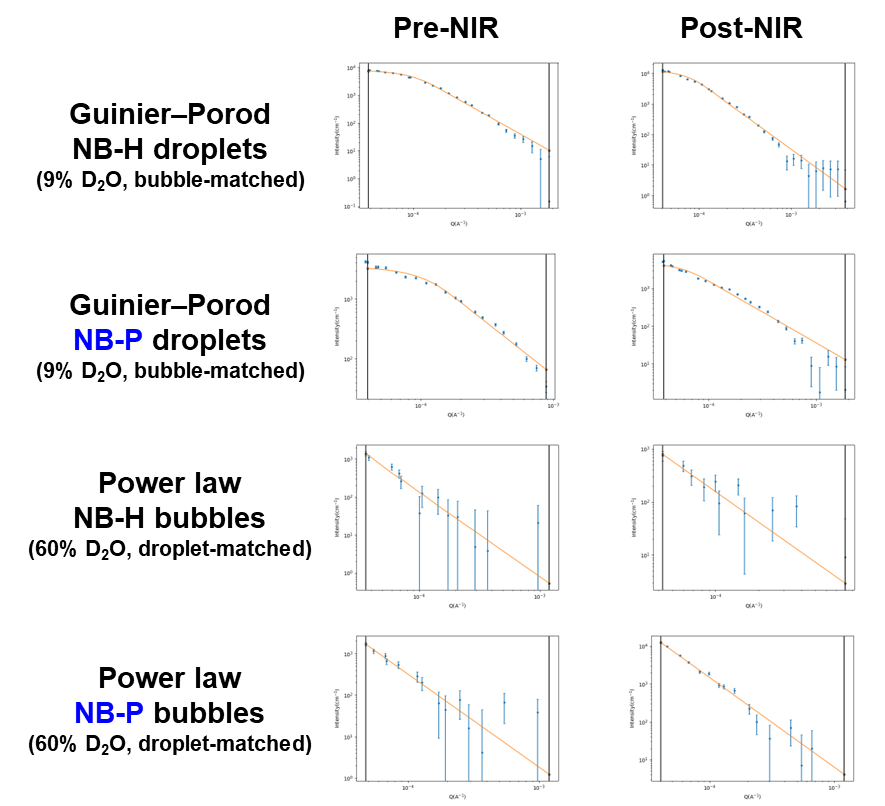
**

**Figure S9.** Plots showing the Guinier–Porod and power law model fits of USANS data from NB-H (PFH) and NB-P (PFP) in 9% and 60% D_2_O before and after NIR illumination (400 mW cm^-2^). Note that the scattering intensity from bubbles (samples at 60% D_2_O: NB-H (pre- and post-NIR) and NB-P (pre-NIR)) are close to background and the model fitting might not be reliable.

**Table S4.** Fitting parameters from the Guinier–Porod model and power law fitting of NB-H and NB-P droplet dispersion in bubble-matched and droplet-matched media at different stages of NIR illumination (400 mW cm^-2^). Parameters obtained correspond to model fitting in Figure S9.

| **Sample** | **Parameter** | **Pre-NIR*** | **Post-NIR*** |
| --- | --- | --- | --- |
| NB-H droplets  (9% D_2_O, Guinier–Porod**) | Rg (nm) | 1390 (22) | 1874 (31) |
|  | Porod | 3.17 (0.03) | 3.20 (0.02) |
|  | χ2 | 5.01 | 5.65 |
| NB-P droplets  (9% D_2_O, Guinier–Porod**) | Rg (nm) | 1280 (28) | 2164 (67) |
|  | Porod | 2.73 (0.03) | 2.64 (0.02) |
|  | χ2 | 7.31 | 12.235 |
| NB-H bubbles  (60% D_2_O, Power law) | Power law*** | 3.22 (0.23) | 1.91 (0.29) |
|  | χ2 | 0.97 | 1.02 |
| NB-P bubbles  (60% D_2_O, Power law) | Power law*** | 2.24 (0.19) | 2.36 (0.05) |
|  | χ2 | 1.98 | 1.98 |

*Values in parentheses are the SasView error values for the fitted parameters.

**Fixed parameter (Guinier–Porod model): s = 0 (3D globular objects, such as spheres).

***Note that the scattering intensity from bubbles (samples at 60% D_2_O: NB-H (pre- and post-NIR) and NB-P (pre-NIR)) are close to background; parameters from the model fitting might not be reliable and lack meaningful interpretation.


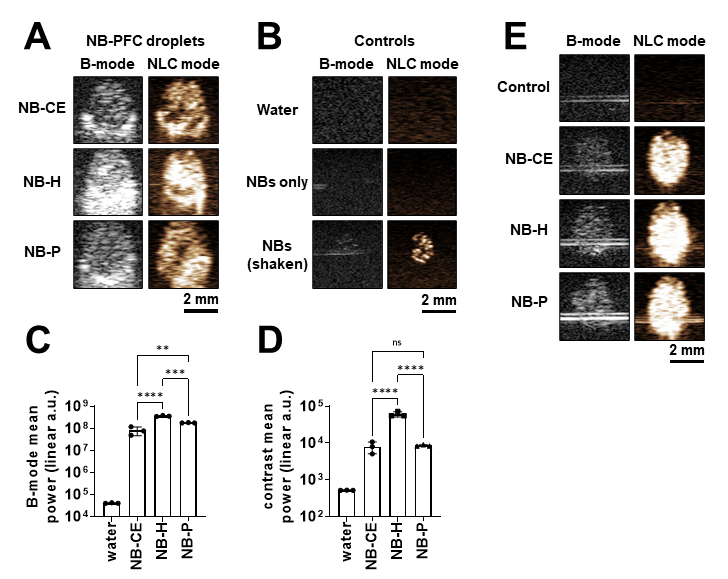


**Figure S10**. Representative B-mode and NLC mode ultrasonograms of tissue-mimicking phantoms containing (**A**) PDA NB-stabilized PFC emulsions (NB-CE = PFCE, NB-H = PFH, and NB-P = PFP core) and (**B**) controls (water, NBs only = freshly sonicated, bubble-less PDA NBs, and shaken NBs = freshly sonicated, bubble-containing PDA NBs). Comparison of contrast from (**C**) B-mode and (**D**) NLC mode ultrasonograms of sample-containing tissue-mimicking phantoms. Data is presented as mean ± SD from three independent experiments (*n* = 3) with at least 20 analyzed frames per experiment; ns = no significant differences, ***p* < 0.01, ****p* < 0.001, *****p* < 0.0001 by one-way ANOVA, followed by Tukey’s multiple comparison test between all PDA NB-stabilized PFC emulsions. (**E**) Representative B-mode and NLC mode ultrasonograms of tissue-mimicking phantoms containing PDA NB-stabilized PFC emulsions rapidly injected into the phantom wells, demonstrating no obvious differences in contrast regardless of sample type. Scale bar = 2 mm. Imaging was conducted in NLC mode, which simultaneously provides a B-mode view and a contrast imaging view at low transmit power (6–10%), theoretical peak rarefactional pressure (1.42 MPa) and mechanical index (0.24).

**
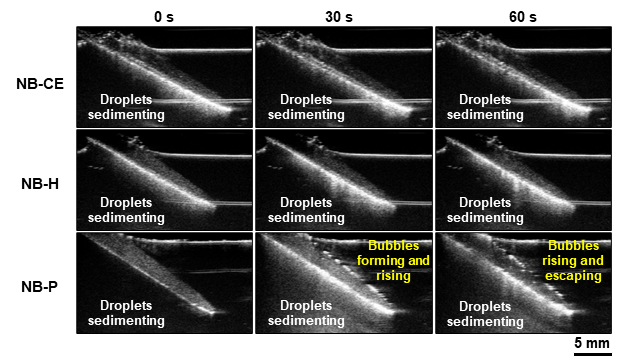
**

**Figure S11**. Representative B-mode mode ultrasonograms of tissue-mimicking phantoms containing PDA NB-stabilized PFC emulsions over a 60-s observation period. NB-CE = PFCE, NB-H = PFH, and NB-P = PFP core. Bubble formation (acoustic droplet vaporization, ADV) was only observed in NB-P droplets. Scale bar = 5 mm. Imaging was conducted in B-mode at maximum transmit power (100%) with free-field values for peak rarefactional pressure = 4.48 MPa and mechanical index = 0.77


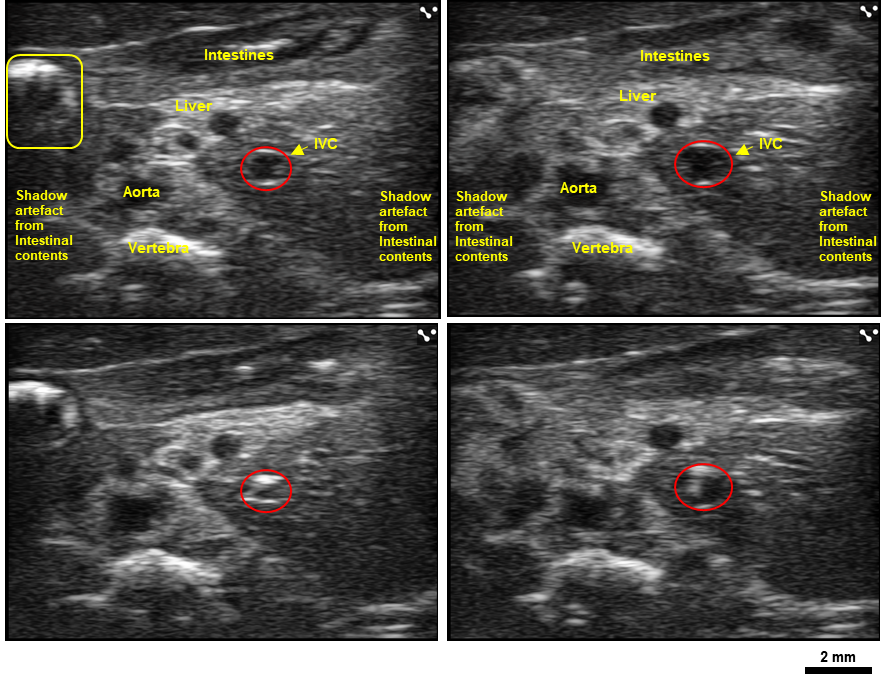


**Figure S12.** Ultrasonograms of the same abdominal region of a mouse in transverse plane view. The top panels are images from different stages of peristalsis before contrast injection. When observing intestinal peristalsis on ultrasound imaging, the movement appears as rhythmic, wave-like contractions along the intestinal walls. The *top left* image shows intestinal gas contents (highlighted by a yellow box and the bright white line above the annotation of intestine), creating a shadow and obscuring the imaging of structures beneath. The *top right* image shows the intestinal content being moved out of view, due to the tightening and then relaxing of the intestinal muscles. In the bottom panel, we have included the images at the respective peristalsis stages after intravenous injection of contrast agents. Acoustic signals can be visualized clearly within the inferior vena cava (highlighted by the red circles). Scale bar = 2 mm.

**Captions for the Supporting Videos**

**Supporting Video 1.** B-mode ultrasonogram of the abdominal region of a PBS control-injected mouse (baseline) in transverse plane view clearly showing that the inferior vena cava, aorta, small mesenteric artery, and other vascular regions are not obscured by the surrounding tissues.

**Supporting Video 2.** B-mode ultrasonogram of the abdominal region of a NB-H-injected mouse in transverse plane view clearly showing strong acoustic signals withing the inferior vena cava, unobscured by the surrounding tissues.
